# IFSEMC 2022: International Festival of Sports, Exercise & Medicine Conference: A joint conference between SASMA, LTMIC, PASHDA and SASB

**DOI:** 10.17159/2078-516X/2022/v34i1a14886

**Published:** 2022-01-01

## Background

Hockey has a high potential for sports related concussion injuries due to intrinsic and extrinsic factors associated with the rules and equipment of the game. This is despite hockey being classified as a non-contact sport. Symptoms of concussion are diverse and may have a delayed presentation contributing to a high number of sports related concussions that are overlooked and not detected. Undiagnosed concussion injuries may result in adverse health complications and contribute towards long-term neurological conditions. Multiple concussion injuries and sub concussive impacts may have a cumulative effect or threshold dose effect contributing to neurodegenerative diseases. Concussion injury protocols are imperative to improve the detection, management, and outcomes of sports related concussion injuries. However, hockey concussion injury protocols are insufficient compared to other sports such as rugby.

## Methodology

This study was a partially mixed sequential dominant status design (QUANT qual), divided into two phases. In phase one a modified RoCKAS-ST questionnaire was conducted with hockey players and officials. In phase two a focus group discussion with umpires and interviews with coaches were conducted.

## Results

Of 101 field hockey players 18,8% were diagnosed with a sports related concussion between March 2018 and March 2022. Of this population 35.6% reported having a concussion before March 2018. Injuries to the shoulder, neck, head, and face were reported as: 98 stick related injuries; 102 ball related injuries; 187 collision related injuries. However, only 20 of these reported injuries resulted in a concussion suggesting that a large number of concussion injuries were overlooked, undetected or not reported at the time of injury. Of those players diagnosed with a concussion, 27,7% continued to play following the injury either because it was an important game or the injury severity was not detected. Coaches and umpires recognised that potential concussion incidents required educational intervention to improve on field diagnosis and management.

## Conclusion

From the total number of reported hockey related injuries (n=387), only 20 (5,2%) were identified as concussion. This could indicate a gap in the knowledge regarding concussion injury detection and on field management. It is imperative that concussion protocols are developed to support player welfare

