# Supplementary material for: IFSEMC 2022: International Festival of Sports, Exercise & Medicine Conference: A joint conference between SASMA, LTMIC, PASHDA and SASB
Source: S Afr J Sports Med. 2022 Jan 1;34(1):v34i1a14886. doi: 10.17159/2078-516X/2022/v34i1a14886 (PMC9924603; doi:10.17159/2078-516X/2022/v34i1a14886)
Supplement: Related texts

# Abstract: A2: CHRONIC DISEASE AND ALLERGIES ARE ASSOCIATED WITH ILIOTIBIAL BAND SYNDROME (ITBS) IN DISTANCE RUNNERS: A CROSS-SECTIONAL STUDY IN 76,654 RACE ENTRANTS – A SAFER STUDY

**Published:** 2022-01-01

**Authors:** Jandre V. Marais, Audrey Jansen van Rensburg, Martin P. Schwellnus, Catharina C Grant, Esme Jordaan, Pieter Boer

**Affiliations:** 1 Section Sports Medicine, Faculty of Health Sciences, University of Pretoria, Pretoria, South Africa; 2 Sport, Exercise Medicine and Lifestyle Institute (SEMLI), Faculty of Health Sciences, University of Pretoria, Pretoria, South Africa; 3 Emeritus Professor of Sport and Exercise Medicine, Faculty of Health Sciences, University of Cape Town, South Africa; 4 International Olympic Committee (IOC) Research Centre, South Africa; 5 Biostatistics Unit, Medical Research Council, South Africa; 6 Statistics and Population Studies Department, University of the Western Cape, South Africa; 7 Department of Human Movement Science, Cape Peninsula University of Technology, South Africa

## Background

Iliotibial Band Syndrome (ITBS) is a common gradual onset running-related injury affecting the lateral side of the knee in distance runners. Risk factors associated with ITBS are sparsely researched. This cross-sectional study aims to identify the risk factors associated with ITBS in distance runners that entered the 21.1km and 56km Two Oceans Marathon races (2012–2015).

## Methodology

In total, 76654 runners (71.8%) of the 106743 race entrants who completed the online pre-race medical screening questionnaire, consented to the study. Among them, 60635 were non-injured, and in 1314 runners an ITBS injury was verified by a health care professional. Risk factors associated with ITBS were explored using uni- & multiple regression analyses: demographics (race distance, sex and age groups), training/running variables, history of existing chronic diseases (including a composite chronic disease score) and any allergy history. Prevalence (%, 95%CI) and prevalence ratios (PR) are reported.

## Results

The 12-month period prevalence of ITBS in marathon runners was 1.58% (1.49–1.68). Independent risk factors (adjusted for sex, age group and race distance) associated with a history of ITBS were a higher chronic disease composite score (PR=2.38 times increased risk for every two additional chronic diseases; p<0.0001) and a history of allergies (PR=1.90; p2; univariate analysis) significantly associated with a history of ITBS were: any GIT disease (PR=3.11; p<0.0001); any haematological/immune disease (PR=2.79; p=0.0038); any kidney/bladder disease (PR=2.56; p=0.0002); any nervous system/psychiatric disease (PR=2.25; p<0.0001); any respiratory disease (PR=2.23; p<0.0001); any symptoms of CVD (PR=2.16; p=0.0106). A significantly lower prevalence of a history ITBS injury is reported for runners with more years of recreational running (PR=0.94, a 6% decrease in risk for every 5-year increase in running; p=0.0009) and a slower average running speed (PR=0.98, a 2% decrease in risk for every 1 km/hr decrease in running speed; p=0.0066).

## Conclusion

The novel independent risk factors associated with a history of ITBS in distance runners are an increased number of chronic diseases and a history of allergies. Identifying athletes at higher risk for ITBS can guide healthcare professionals in their prevention and rehabilitation efforts.

# Abstract: A3: A COMPARATIVE STUDY INVESTIGATING THE FIFA11+ INJURY PREVENTION PROGRAMMES FOR IMPLEMENTATION IN RECREATIONAL SOCCER, TENNIS AND CRICKET

**Published:** 2022-01-01

**Authors:** David Kopping, Dylan Bennett, Habib Noorbhai

**Affiliations:** 1 Department of Sport and Movement Studies, Faculty of Health Sciences, University of Johannesburg, South Africa

## Background

Sports injuries have long since plagued professional and recreational athletes alike. The risk of injuries sustained by recreational athletes may be reduced through effective Injury Prevention Programme’s (IPP’s) performed prior to exercise. The FIFA 11+ and FIFA 11+ Shoulder (11+S) are IPP’s that were designed and have been effectively implemented in professional soccer. However, a gap in the literature exists as standardised IPP’s designed for tennis and cricket are a scarce resource. This study aimed to identify whether these IPP’s would be efficacious in reducing the risk of injury for recreational athletes across multiple sporting codes in South Africa.

## Methodology

An online questionnaire was distributed to recreational athletes. Valid responses were recorded (n = 64). Chi square tests were conducted to determine the correlation between coaches discussing injury prevention techniques and the participant’s injury outcome. Chi-square tests were also conducted to determine whether a relationship existed between a participant’s warm-up routine and pain experienced during sports participation.

## Results

Over half (52%) of the participants reported having a sports-related injury within three months prior to their questionnaire completion. Furthermore, a combined 34% of participants reported experiencing pain whilst performing their respective sport. A resounding 86% of the participants were unfamiliar with the FIFA 11+ IPP, yet 48% of the participants reported always warming up prior to exercise. The ankle joint was most prevalently injured in soccer players (91%) while the knee/patella was the most prevalent injury site (67%) in tennis players. Surprisingly, 42% of cricketers reported having neurological symptoms (numbness or tingling) down one or both legs. Additionally, 25% of cricketers also reported experiencing lower back pain.

## Conclusion

This study’s questionnaire responses revealed that recreational players do perform a warm-up routine prior to exercise, yet sustained injuries occur regardless. Therefore, the FIFA IPP’s should be considered to standardise warm up programmes for recreational sports participation in South Africa.

# Abstract: A4: INJURY AND ILLNESS ASSESSMENT AT THE 2016 FOUR-DAY HIGH SCHOOL NETBALL TOURNAMENT AT WATERKLOOF: A CROSS-SECTIONAL STUDY

**Published:** 2022-01-01

**Authors:** Elene Lourens, Audrey Jansen van Rensburg, Tanita Botha, Dina Christa Janse van Rensburg

**Affiliations:** 1 University of Pretoria

## Background

Netball is growing in popularity with increasing numbers of participants at the school level. This expands the talent pool but may lead to more competitive play and more netball-related medical encounters. This study aimed to determine the period prevalence, incidence, type and diagnosis of injuries and illnesses in female netball players during the 2016 four-day Waterkloof High School Netball tournament.

## Methodology

This cross-sectional observational study involved all participating female youth netball players (n=875) from 41 high schools and 125 teams (u/14 – u/19). Players suffering from an injury or illness were assessed in the medical tent by a medical practitioner who completed an injury/illness data collection sheet. Main outcome measures included: 1) injury and illness period prevalence (PP: %), 2) injury incidence (I: per 1000 match player-hours) for anatomical region, body area, tissue type, pathology type and specific diagnosis, and 3) illness incidence (I: per 1000 match player-days) for organ system, region, symptom cluster and aetiology of illness.

## Results

In total, 239 players sustained 262 match injuries (PP=27.31%). The incidence of all injuries was 48.13. Lower limb injuries were the most common (I=30.49). The most frequent body area involved was the ankle (I=12.12) followed by the knee (I=9.00) and the foot (I=5.69). Ligament/joint capsule was the most common tissue type involved (I=13.59). Joint sprains (I=13.59) are attributed to the highest pathology type, primarily presenting as ankle sprains (I=10.84) of the lateral ligament (I=6.43). A total of 42 players contracted 42 illnesses (PP=4.80%). The incidence of all illnesses was 12.0. The dermatological system (I=3.71) was the most common system affected, followed by the respiratory system (I=1.71). Itchy lesions were the most frequent symptom reported (I=3.43). Most illness encounters are associated with exercise-related causes.

## Conclusion

The lower limb specifically the ankle was the most frequently affected body area. Ligament/joint capsule was the most common tissue type involved, lateral ankle sprains were the most common diagnosis. The dermatological system was most commonly affected followed by the respiratory system. Injuries were more prevalent than illness. Knowledge of injury and illness epidemiology will enable the development of preventative measures to reduce injury/illness patterns at youth netball tournaments.

# Abstract: B2: AN EXPLORATION OF SUPPORT SYSTEMS FOR ELITE ATHLETES BY SOUTH AFRICAN NATIONAL SPORT FEDERATIONS

**Published:** 2022-01-01

**Authors:** Solomon Mthombeni, Habib Noorbhai, Yoga Coopoo

**Affiliations:** 1 Sport and Movement Studies, Faculty of Health Sciences, University of Johannesburg

## Background

Athletic success at major events such as the Olympic Games is of significant importance and value to elite athletes, sports coaches, various stakeholders and nations. The purpose of the study was to examine the availability of elite sport support systems by the South African National Sports Federations (NSFs) and whether support systems were extended and accessible to elite athletes from historically disadvantaged areas.

## Methodology

The paper was a descriptive study in which analytical methods were employed. A total of 21 NSFs with Olympic sporting codes participated in the study whereby they responded to a survey questionnaire in line with sports policy factors leading to sporting success (SPLISS, 2015) on support systems for elite athletes. Descriptive statistical analyses were conducted using Statistical Package for Social Sciences (SPSS version 27.0) and arranged into customised tables of frequencies.

## Results

In terms of the overall availability of support systems for elite sports by NSFs, the majority of federations reported overall insufficiencies in the following pillars of support (Overall vs. Historically disadvantaged areas); financial resources 11.6±8.8 vs. 9.8±10.5 (61.9% vs. 66.7%), scientific research support services 11.8±7.1 vs. 7.8±9.1 (52.4% vs. 61.9%), followed by post-sport career support 11.8±6.6 vs. 9.6±9.4 (47.6% vs. 66.7%), and sports facilities 14.2±9.2 vs. 9.6±9.4 (23.8% vs. 66.7%). In terms of elite sport support systems, NSFs reported moderate to high sufficiency of the following support systems; coach provision and development programmes 14.2±9.2 (90.5%), organisational structure and sports policies 14.2±9.2 (81%), and opportunities for international competitions 14.2±9.2 (81%).

## Conclusion

The insufficiency of support systems was worse for elite athletes from historically disadvantaged areas in all the nine pillars of support systems, with provision for sport infrastructure being among the highest insufficiencies reported by NSFs. The NSFs reported having sufficient organisational and sport policy structures in place, as well as provision for access to international competitions. However, more provision is required to ensure that organisational structure and sport policies are more inclusive towards athletes from historically disadvantages areas. These additions in support systems may contribute ultimately to better outcomes in performance, and broadening the pool of athletes performing for South Africa.

# Abstract: B3: RHYTHMIC MOVEMENT AS ALTERNATIVE TRAINING METHOD FOR RUGBY PLAYERS

**Published:** 2022-01-01

**Authors:** Jocelyn Solomons

**Affiliations:** 1 Stellenbosch University

## Background

The inclusion of other non-traditional approaches to training has become more popular within rugby conditioning. Rhythmic movement, also referred to as “dance”, involves the execution of different motor skills, the integration and sequencing of actions between limbs, timing and spatial precision. It requires performing movement tasks to auditory rhythmic patterns and as a multifaceted activity, it depends on a large number of elements with direct and indirect effects on the physiology and physical attributes of a player. In terms of rugby conditioning, the common belief dictates that fitness or conditioning elements should be developed through focused, isolated training blocks. The technical, tactical and physical conditioning for rugby has primarily consisted of traditional, rugby-based approaches to training as indicated by the majority of current research. However, in order for rugby coaches and specialist coaches to gain a competitive edge over opposing teams, they need to find new innovative ways to adapt their training methods and programmes in order to accommodate the changes to the profile of the game. The primary aim was to investigate the effect of a rhythmic movement intervention on selected bio-motor skills of rugby players in the Western Province Rugby Union Academy

## Methodology

The current study was based on a crossover experimental design. A crossover trial involves two treatments, which are administered consecutively to all the participants recruited for the study. The main purpose served by this study design was to provide a basis for separating treatment effects from period effects and to establish whether the intended outcome(s) of the intervention materialised. This separation was achieved by calculating the treatment effects separately in two sequence groups, which is done by the process of randomisation. In this design, pre-post changes in the experimental group were directly compared to changes in the control group to indicate the effects of the intervention. Crossover trials require a washout period to ensure that baseline data are comparable. The reversibility of a treatment effect is a prerequisite for applying a crossover design and determines the length of the washout period. Academy rugby players (N = 54) from the Western Cape, South Africa (age 18 0.81 years; height 1.76 0.69 cm; weight 76.77 10.69 kg), were conveniently selected to participate in this study.

During the pre-and post-test, all the participants were tested on various fitness elements in a field testing order. The rhythmic movement programme was conducted and choreographed by the primary researcher who is a professional dancer and choreographer. In order to compile the intervention, the primary researcher looked at the most common movement patterns and exercises of rugby players by studying match footage. The intervention consisted of 32, 60-minute sessions over a period of 16 weeks (2 × 8 weeks). These sessions were part of their weekly planning and were not extra sessions. Each session started with a 10-minute progressive aerobic endurance rhythmic movement routine as a warm-up. The warm-up was followed by 45 minutes of learning new rhythmic movement exercises and repeating them to music, which concluded with a 5-minute cool-down which involved progressive stretching.

The data was analysed using descriptive statistics (standard deviations and means). A series of one-way ANOVA with post hoc LSD t-tests were used to examine between-group (TCA versus CTB group) differences. Statistical significance at 5% (p 0.05) were highlighted (in cases where p = 0.06).

## Results

Results indicated a statistically significant improvement (p < 0.05) in agility2 (p = 0.06), power2 (p = 0.05), local muscular endurance1 (p = 0.01) & 3 (p = 0.01) and dynamic balance (p < 0.01). Likewise, forwards and backs also showed statistically significant improvements (p < 0.05) per positional groups.

## Conclusion

Therefore, a rhythmic movement intervention has the potential to improve rugby-specific bio-motor skills and improve positional-specific skills should it be designed with positional groups in mind. Future studies should investigate, not only the effect of rhythmic movement on improving specific rugby bio-motor skills but the potential of its application as an alternative training method during off-season (or detraining phases) or as a recovery method.

# Abstract: B4: THE INTER- AND INTRA-RATER RELIABILITY OF THE MOVEMENT COMPETENCY SCREEN IN EXPERIENCED AND INEXPERIENCED RATERS

**Published:** 2022-01-01

**Authors:** Kristian Myburg, Bradley Hornsey, Christiaan Jordaan, Helen Bayne

**Affiliations:** 1 Department of Physiology and Sport, Exercise Medicine and Lifestyle Institute, Faculty of Health Sciences, University of Pretoria

## Background

Movement screening is used to predict future performance and identify individuals at increased risk of injury. The Movement Competency Screen (MCS) may be the preferable tool for athletes as it assesses seven fundamental movement patterns at two different loading levels and makes use of video cameras, purportedly to enhance the reliability of rating. Movements are rated subjectively based on specified criteria and, therefore, experience in rating the test may influence scoring. The aim of the study was to assess inter- and intra-rater reliability in a group of diversely experienced sports scientists in conducting the MCS with adolescent athletes.

## Methodology

Three experienced (>2 years in conducting the test) and three inexperienced (<1 year) raters scored 10 MCS videos on two occasions separated by three weeks. Each movement was rated on a scale of 1 to 5, with a possible total of 50. Kendall’s coefficient of concordance (W) was used to assess inter-rater reliability for each movement and the total score. Intra-rater reliability was assessed using the Kappa statistic for each movement and the intra-class correlation coefficient (ICC) for the total.

## Results

Inter-rater reliability was similar in both groups of raters, with a moderate agreement for most of the movements (mean W; Experienced: 0.58 on day 1, 0.59 on day 2; Inexperienced: 0.58 on day 1, 0.62 on day 2). However, the intra-rater reliability was substantially higher in the experienced raters (ICC for total scores of 0.64 compared to 0.25 in the inexperienced), demonstrating a better consistency across the two tests.

## Conclusion

Inter-reliability was only moderate for both the experienced and inexperienced raters. Care should therefore be taken when multiple raters are involved in assessing groups of athletes. Intra-rater reliability appears to be affected by the testing experience. It is therefore recommended that thorough training is emphasised and that practitioners working in a team should regularly collaborate on implementing the MCS to improve agreement between raters.

# Abstract: B5: THE EFFECTS OF TIME OF DAY ON ISOKINETIC TORQUE AND POWER DURING KNEE EXTENSION AND FLEXION: A PILOT STUDY

**Published:** 2022-01-01

**Authors:** Motheo Moroane, Jonathan Davy

**Affiliations:** 1 Department of Human Kinetics and Ergonomics, Rhodes University

## Background

Time of day influences both physical and cognitive performance. This influence may have further implications for when coaches or clinicians schedule assessments involving isokinetic dynamometry when attempting to quantify strength and power. The aim of this study, therefore, was to determine the effects of time of day on isokinetic measures of strength and power.

## Methodology

Through a repeated measures experimental design, this study assessed the time of day effects on isokinetic peak torque, average peak torque, and average variables during knee extension and flexion at angular velocities of 60°/s and 180°/s using the Isokinetic Dynamometer System 4 Pro. 10 healthy student participants completed three testing sessions over six days. The three experimental conditions were in the morning (08h00–09h00), afternoon (13h00–14h00), and evening (18h00–19h00). Five repetitions of isokinetic knee extension and flexion were performed at each velocity, with a 60-sec break between testing velocities. A one-way analysis of variance was applied to compare the three times of day with a Tukey post hoc test applied when relevant.

## Results

During knee extension at 60°/s, peak torque (p=0.01) and average peak torque (p=0.03) were significantly higher in the evening relative to other times of the day. No other statistically significant differences were reported for either knee extension at 60°/s or knee extensions at 180°/s and knee flexion at 180°/s. Although no significant differences were found for these variables, there was a trend towards greater torque and power variables in the afternoon and evening for knee extension and knee flexion.

## Conclusion

The findings indicate that time of day does have an impact on torque produced at lower velocities, but not higher velocities. It is therefore crucial for researchers, practitioners, and high-performance specialists to factor these effects when using isokinetic dynamometry to ensure that accurate measures are obtained, particularly when testing for maximum torque production during isokinetic dynamometry

# Abstract: C1: DETECTION OF DOPING USING OCULAR-MOTOR DECEPTION TESTING

**Published:** 2022-01-01

**Authors:** Belinda Adigun, Jeroen Swart

**Affiliations:** 1 Research Centre for Health through Physical Activity, Lifestyle and Sport (HPALS), Dept of Human Biology, Faculty of Health Sciences, University of Cape Town

## Background

The manipulation of haemoglobin mass has been used by athletes to enhance performance. The adaptive biological passport assesses haematologic markers for patterns indicating a high probability of doping. Athletes use counter-measures to subvert testing, thus requiring a detection method independent of haematologic variables. Ocular-motor deception testing (OMDT) utilises automated pupillometry and eye movement tracking to detect deception in subjects. The objective of this study is to investigate the sensitivity and specificity of OMDT in the detection of doping, as an independent marker.

## Methodology

This sham study is designed to replicate a typical 12-week training season for athletes leading up to a major competition. We employed a randomised, single-blinded, control study. Twenty subjects were randomised into a control or doping group and provided with a standard 12-week cycling training program. Subjects in the doping group received placebo capsules under the guise of a performance-enhancing substance (PES), for the duration of the training program. OMDT was performed at weeks 1, 8, 13 and 20 to establish the washout period. All subjects were monetarily incentivised to evade detection. A repeated measures ANOVA, and post hoc Bonferroni analysis was conducted to compare the two groups over time and between each time point.

## Results

The two groups responded differently over time (p=0.0251; F(3,9)=5.071). OMDT testing performed on week 8 (during the consumption of placebo) resulted in (Mean ± SD (Range)) scores of 79.75 ± 10.8 (64 – 88) for the control group and 35.8 ± 15.5 (17–49) for the doping group respectively. At a threshold value of 50, all of the subjects in the doping and control groups recorded below and above the threshold respectively. However, data from before the commencement, and after the cessation, of PES did not result in similar uniform outcomes.

## Conclusion

Results suggest that OMDT can reliably detect deceptive behaviour while subjects are taking PES. However, 8 weeks after treatment is discontinued, OMDT no longer discriminates between subjects in the doping and control groups. Overall, results suggest that OMDT could potentially be used as an indirect detection method to identify athletes for further investigation into potential drug use.

# Abstract: C2: CONCURRENT LOW CARBOHYDRATE, HIGH FAT DIET WITH/WITHOUT PHYSICAL ACTIVITY DOES NOT IMPROVE GLYCEMIC CONTROL IN TYPE 2 DIABETICS

**Published:** 2022-01-01

**Authors:** Gerrit J Breukelman, Albertus K Basson, Trayana G Djarova, Cornelia J Du Preez, Ina Shaw, Heidi Malan, Brandon S. Shaw

**Affiliations:** 1 University of Zululand, Department of Human Movement Science; 2 University of Zululand, Department of Biochemistry and Microbiology; 3 University of Zululand, Department of Consumer Sciences; 4 Richards Bay, Caredoc

## Background

This study aimed to determine if a low carbohydrate, high fat diet (LCHFD) provides any benefits of glycemic control in patients with type 2 diabetes mellitus, either alone or in conjunction with physical activity.

## Methodology

Type 2 diabetics (n = 39) were assigned into either a concurrent physical activity and LCHFD group (DiExG), LCHFD only group (DietG), or control group (ConG).

## Results

No significant (p > 0.05) changes were observed in glycated hemoglobin (HbA1c), glucose and insulin in either the DiExG (HbA1c: p = 0.592; 8.3% decrease, glucose: p = 0.477; 11.1% decrease and insulin: p = 0.367; 44.1% increase) or DietG (HbA1c: p = 0.822; 0% change, glucose: p = 0.108; 11.0% decrease and insulin: p = 0.976; 4.2% decrease).

## Conclusion

In this study, neither a LCHFD alone nor in combination with a physical activity programme succeeded in eliciting improvements in insulin sensitivity in the type 2 diabetics. As such, adoption of a LCHFD, either alone or in combination with physical activity, should not unequivocally be part of the treatment approach for type 2 diabetics. Furthermore, it should carefully be weighed against the benefits of more balanced dietary and/or physical activity interventions.

# Abstract: C3: VISUAL ABILITIES DISTINGUISH LEVEL OF PLAY IN RUGBY

**Published:** 2022-01-01

**Authors:** Lourens Millard, Brandon S. Shaw, Gerrit-Jan BreukelmanIna Shaw

**Affiliations:** 1 University of Zululand

## Background

Novices in sport possesses similar visual skills to that of experts however, there may be major differences in the magnitude of performance in these skills, with expert athletes only demonstrating superiority in specific vision skills and not all aspects of vision.

## Methodology

The present study compared the performance of Premier League rugby players (n = 40) and First Division rugby players (n = 40) on six specific components of vision, namely; accommodation facility, saccadic eye movement, speed of recognition, peripheral awareness, visual memory, and hand-eye coordination.

## Results

Premier League rugby players performed significantly (p = 0.001) better than the First Division rugby players in five of the six tests. but were found to be similar in visual memory performance (p = 0.810).

## Conclusion

While this study substantiates the proposal that expert athletes, and specifically rugby players, have superior visual expertise to novice athletes, this study also found that this is not the case with all vision skills. The present study’s findings suggest that sport-specific vision testing batteries may be required to distinguish high performers from low performers in the same vein as physical tests are utilised in the selection and recruitment of athletes.

# Abstract: C4: GAMIFICATION FOR BIOKINETICS AND SPORTS SCIENCE: ENHANCING STUDENTS’ CLINICAL SKILLS DURING DISTANCE LEARNING AND POST COVID-19

**Published:** 2022-01-01

**Authors:** Habib Noorbhai, Simone Ferreira

**Affiliations:** 1 Faculty of Health Sciences, University of Johannesburg

## Background

During the COVID-19 pandemic and lockdown levels, it has been a challenge to teach clinical skills and competencies to students due to restrictions on campus and with the influx of students (and their families) becoming infected with the virus. This has propelled universities to become innovative and creative with clinical skills in teaching, learning and assessment.

## Methodology

This teaching approach has been successfully conducted in other countries through the use of simulated-based learning. However, this has not been the ideal option for a student cohort in South Africa due to three main challenges: 1) connectivity issues for certain students, 2) lack of fun and excitement, and 3) lack of infrastructure in the current Biokinetics clinic to enable a telehealth approach. One way in which these challenges have been mitigated is through the adoption of gamification (a 3D game) as a teaching approach. Such an approach has been proven useful in medical teaching and is aligned with the fourth industrial revolution (4IR) imperatives.

## Results

Gamification or game development in teaching is a design-based research approach that includes futuristic development perspectives. In our study, patient case studies and scenarios through gamification have been developed, namely: 1) blood pressure and heart rate, 2) ECG, 3) cycle ergometer test, 4) isokinetic knee flexion/extension, and 5) isokinetic shoulder flexion/extension. These scenarios within the game, factor in the integrations of various lab-based instruments (in 3D) used in Biokinetics practice (examples: the isokinetic machine for orthopaedic rehabilitation, the cycle ergometer for cardiorespiratory screening and fitness, etc). The work-integrated learning (WIL) programme would also be followed and be aligned to these procedures. These procedures have been written as patient scenarios and case studies and have been converted into an interactive gaming solution to assist students with clinical learning, reasoning, skills competencies and assessment of these competencies.

## Conclusion

The game can be accessed via Blackboard (UJ’s learning management system). Thereafter, a virtual reality (VR) approach will be adopted and user-friendly VR devices will be integrated with mobile phones for students to seamlessly utilise and apply. Furthermore, the game will also be deployed as a mobile application on iTunes and GooglePlay.

# Abstract: C5: EXPLORATION OF SAQA CERTIFIED ROCK CLIMBERS’ PERCEPTIONS REGARDING THE CERTIFICATION PROCESS IN SOUTH AFRICA

**Published:** 2022-01-01

**Authors:** Yolanda Stevens, Minette Strauss, Yolanda Stevens, J. Theron Weilbach

**Affiliations:** 1 Leisure Studies

## Background

As individuals engage in progressively more recreational activities, the South African rock climbing industry has shown tremendous growth. The growth brings about numerous safety challenges that specifically relate to skills, experience, and equipment. Due to the limited number of qualified South African guides who specialise in rock climbing as an adventure activity, this research aimed to explore the perceptions regarding the SAQA guiding qualification process to identify any difficulties or hindrances facing rock climbers.

## Methodology

To explore qualified guides’ perceptions, a qualitative descriptive design was implemented, and semi-structured interviews were conducted with five SAQA qualified rock climbing guides in SA. The interview data were analysed and categorised using an inductive content analysis approach.

## Results

From the findings, three themes, namely legality in climbing, fundamentals and climbing culture emerged as themes. It became clear that there is uncertainty regarding the requirements for legal guidance. There are also concerns about the fundamental requirements in the SAQA process, the South African qualification process is different to that of other countries and the perception about the SAQA guiding qualifications can be perceived as inferior. Lastly, the climbing culture contributes to the attitudes climbers have towards the SAQA process. If the correct information about the SAQA qualification is available, the perceptions of the qualification might become more positively persuaded.

## Conclusion

To improve the perceptions towards the SAQA qualification, a single governing body that regulates all training courses and programmes that are available in SA, should be established. Hereby, the qualification can be regulated and monitored in a professional structured manner. This will ensure that adventure guiding in SA is considered as a professional career path from which a person could earn a living and prevent unqualified guides from operating illegally.

# Abstract: D1: THE LINK BETWEEN PHYSICAL ACTIVITY, SEDENTARY BEHAVIOR, DIGITAL HEALTH INTERVENTIONS AND RISK OF TYPE 2 DIABETES AND DIABETES-RELATED HEALTH OUTCOMES: SYSTEMATIC REVIEW AND META-ANALYSIS

**Published:** 2022-01-01

**Authors:** Adiele Dube, M Mathunjwa

**Affiliations:** 1 University of Zululand

## Background

There are more than 4 million people diagnosed with Type 2 diabetes in South Africa, which costs the NHD ZAR 24.5 billion if both diagnosed and undiagnosed patients are considered. This disease significantly increases the risk of cardiovascular disease and reduces the quality of life. However, little is known about the link between physical activity, sedentary behaviour, digital health interventions and risk of Type 2 diabetes and diabetes-related health outcomes. To systematically review the evidence and correlate the effect of physical activity, digital health interventions, sedentary behaviour and risk of Type 2 diabetes and diabetes-related health outcomes.

## Methodology

Electronic databases including Pub Med, MEDLINE, EBSCOT, EMBASE, CINAHL, PsycINFO, SPORT Discus, Science Direct and Web of Science were searched for current and completed interventional trials investigating the effect of either effect of physical activity, digital health interventions, sedentary behaviour and risk of Type 2 diabetes and diabetes-related health outcomes. Search terms included Medical Subject Heading (MeSH) terms and text words.

## Results

Identified were 720 full texts and 12 studies with 450, 769 included. Higher total physical inactivity, sitting time, and sedentary behaviour were associated with a significant risk of obesity (HR=1.27, 95% CI=1.21 – 1.29, p<0.001) and Type 2 diabetes mellitus (HR=1.17, 95% CI=1.03 – 1.25, p<0.001). The increased risk of T2DM was attenuated by a reduction of total daily sitting time and adjusted physical activity but remained significant (HR=1.14, 95% CI=1.02 – 1.16, p<0.001). AOR meta-analysis of the 5 studies showed that higher T2DM rates in women (OR=1.57, 95% CI=1.42 – 1.76, p<0.001) compared to their male counterparts (OR=1.36, 95% CI=1.31 – 1.52, p<0.001) but no differences for T2DM in within age groups.

## Conclusion

Evidence showed that reducing total daily sitting time (i.e. sedentary behaviour) is associated with a reduced risk of T2DM as well as improved glycaemic control in patients already diagnosed with the disease. There is consistent evidence that breaking up sitting time with short, frequent bouts of light-intensity physical activity improves metabolic biomarkers over the course of a single day.

# Abstract: D2: OSTEOARTHRITIS REHABILITATIVE PRACTICES AMONG BIOKINETICISTS AND PHYSIOTHERAPISTS IN SOUTH AFRICA

**Published:** 2022-01-01

**Authors:** Robynne Gilchrist, Aayesha Kholvadia

**Affiliations:** 1 Nelson Mandela University; 2 Biokinetics Association of South Africa; 3 Health Professions Council of South Africa; 4 South African Sports Medicine Association

## Background

A multifaceted condition such as osteoarthritis is ideally suited to the realm of multidisciplinary management which focuses on holistic patient care. Although a biopsychosocial approach to osteoarthritis management has been proposed, there is however a lack of adherence to evidence-based guidelines which is a concern previously found in the South African healthcare system. Therefore, the aim of this study was to evaluate and describe osteoarthritis rehabilitative practices among biokineticists and physiotherapists, thereby identifying current trends in osteoarthritis management.

## Methodology

A descriptive methodology with a cross-sectional study design and a convenience sampling technique was used. The target population consisted of biokineticists and physiotherapists located within the South African public and private healthcare sectors. A self-developed, content-validated, online questionnaire surveyed rehabilitative professionals’ management modalities and patient referral trends.

## Results

Physical exercise (94%) was the most prescribed therapy for osteoarthritis patients among biokineticists and physiotherapists. Almost all biokineticists (89%) and physiotherapists (87%) stated that they refer osteoarthritis patients. Biokineticists (55%) and physiotherapists (50%) would most likely refer an osteoarthritis patient to a specialist medical practitioner. Notably, the majority of biokineticists (55%) also indicated that they would most likely refer an osteoarthritis patient to a physiotherapist.

## Conclusion

Understanding the various rehabilitative practices and the unique role of each profession could guide practitioners regarding ways to create and promote an environment conducive to a holistic approach to osteoarthritis management facilitated by referral systems that fit with the theory of a biopsychosocial approach.

# Abstract: D3: TEAM-BASED APPROACH TO OSTEOARTHRITIS MANAGEMENT: VIEWPOINTS OF BIOKINETICISTS AND PHYSIOTHERAPISTS

**Published:** 2022-01-01

**Authors:** Robynne Gilchrist, Aayesha Kholvadia

**Affiliations:** 1 Nelson Mandela University; 2 Biokinetics Association of South Africa; 3 Health Professions Council of South Africa; 4 South African Sports Medicine Association

## Background

There are challenges within healthcare systems when providing the appropriate continuum of care to address the complexity of patients with osteoarthritis. The range of outcomes and multitude of symptoms and etiologies associated with osteoarthritis calls for a team-based approach for optimal management. Rehabilitative professionals are encouraged to collaborate with professionals from diverse health professions to deliver quality patient care. However, there is little published evidence exploring their perceptions of a team-based approach. The purpose of this study was to determine and describe the viewpoints of biokineticists and physiotherapists regarding a team-based approach to osteoarthritis management in rehabilitative medicine.

## Methodology

A descriptive methodology with a cross-sectional study design and a convenience sampling technique was used. The target population consisted of biokineticists and physiotherapists located within the South African public and private healthcare sectors. A self-developed, content-validated, online questionnaire surveyed rehabilitative professionals’ perceptions of a team-based approach to osteoarthritis management.

## Results

Solo practices were the most popular description for both physiotherapists (33%) and biokineticists’ (55%) current practice setting. Overall communication among team members was viewed as dissatisfactory by physiotherapists (36%) and biokineticists (43%). Respectively, 69% and 54% of the physiotherapists and biokineticists felt adequately educated on the scope of practice of various healthcare professions involved in osteoarthritis management. Forty-three per cent of participating rehabilitative professionals indicated that they had not been exposed to interprofessional education.

## Conclusion

There are equal perceptions among biokineticists and physiotherapists regarding a team-based approach to osteoarthritis management. Awareness of South African rehabilitative professionals’ experiences of a team-based approach could guide best-practice recommendations to enhance organised teamwork to promote service delivery and quality care for the osteoarthritis patient.

# Abstract: D4: PHYSICAL ACTIVITY AND QUALITY OF LIFE OF IN PATIENTS WITH FIBROMYALGIA – A PILOT STUDY

**Published:** 2022-01-01

**Authors:** Lynn Smith

**Affiliations:** 1 University of Johannesburg

## Background

Patients with fibromyalgia syndrome (FMS) experience a lower quality of life, largely affecting their social functioning and mental health. Physical function has been linked to improvements in self-efficacy and social behaviour and lowered anxiety and depression. The purpose of this research was to determine the relationship between physical activity, quality of life and the impact of FMS on diagnosed individuals. The objectives of the study were to determine the likelihood of FMS patients participating in physical activity regularly, to determine the relationship between physical activity and overall quality of life, and to determine the relationship between quality of life and the impact of FMS on these patients.

## Methodology

This study was cross-sectional, and descriptive and quantitative data were collected. Thirty-eight patients with FMS completed an online questionnaire of four components. The four main components included Demographics, the Fibromyalgia Impact Questionnaire (FIQR), Short Form-36 (SF-36), and the Global Physical Activity Questionnaire (GPAC). The analysis included descriptives, correlations and statistical significance. The significance levels were set at p ≤ 0.05 and p ≤ 0.0167.

## Results

The results exhibited a high FIQR score and a low SF-36 score, suggesting a negative impact on participants’ quality of life. Results do not imply that physical activity had an influence on the FIQR and SF-36 scores, however, little to no statistical evidence exists to support this.

## Conclusion

It was concluded that although FMS does have a direct impact on quality of life, the relationships between physical activity, sedentary time, the impact of FMS and quality of life were inconclusive. A need for further research on this topic has been identified.

# Abstract: E1: PREVALENCE AND INCIDENCE OF INJURIES AMONG FEMALE CRICKET PLAYERS: A SYSTEMATIC REVIEW AND META-ANALYSES

**Published:** 2022-01-01

**Authors:** Jolandi Jacobs, Benita Olivier, Muhammad Dawood, Nirmala Perera

**Affiliations:** 1 University of the Witwatersrand; 2 Sefako Mokgatho Health Sciences University; 3 Australian Insititute of Sport

## Background

Cricket, a bat-and-ball sport, is becoming popular among women of all ages and abilities worldwide. However, cricket participation carries a risk of injury. Injuries negatively affect sports participation, performance, and short- and long-term health and well-being. Injury prevention, therefore, is the key to safe, long-term cricket participation as a physical activity goal.

## Methodology

A systematic review and meta-analyses were conducted according to the JBI and PRISMA 2020 guidelines. Databases (including grey literature databases) were systematically searched from inception to August 2021. Full-text articles that met the inclusion criteria were critically appraised using JBI-tools, and were extracted and synthesized in the narrative summary and tabular forms. Three meta-analyses were conducted and heterogeneity was assessed using the I^2^ statistic and the random effects model.

## Results

Of the 4256 studies identified, 23 studies met the inclusion criteria. The risk of bias was low for 21 studies. The injury incidence rate for elite cricket was 71.9 (SE 21.3, 95% CI 30.2–113.6) injuries per 1000 player hours, the time-loss injury incidence rate was 13.3 (SE 4.4, 95% CI 4.6–22.0) injuries per 1000 player hours, and non-time-loss injury incidence rate was 58.5 (SE 16.9, 95% CI 25.6–91.7) injuries per 1000 player hours. The injury prevalence proportions for community to elite cricket was 65.2% (SE 9.3, 95% CI 45.7–82.3) and injury prevalence proportions for community cricket was 60% (SE 4.5, 95% CI 51.1–68.6). The injury incidence proportions for community cricket was 5.6 (SE 4.4, 95% CI 0.1–18.3) injuries per 10,000 participants. Elite cricket players were more frequently injured than community cricket players. The most prevalent body regions injured were the shoulder, knee, ankle, foot, and toes, and most were sustained by fast bowlers. Injuries to the hand, wrist, and fingers had the highest incidence and were most sustained by fielders.

## Conclusion

The study’s findings can help stakeholders make informed decisions about cricket participation by informing and implementing strategies to promote cricket as a vehicle for positive public health outcomes. This review also identified gaps in the available evidence base and addressing these through future research would enhance women’s cricket as a professional sport.

# Abstract: E2: AN EVALUATION OF HOW CRICKETERS ARE COACHED TO BAT FROM JUNIOR CRICKET

**Published:** 2022-01-01

**Authors:** Habib Noorbhai, Zaid Kola, Jordan Stringer

**Affiliations:** 1 Faculty of Health Sciences, University of Johannesburg

## Background

Cricket batters have been taught certain batting techniques at different levels of the game by a variety of cricket coaches. A number of questions have been raised about the various aspects of batting that were taught to players since they started playing cricket from junior level. Since previous research investigated the teachings of coaches, the aim of this study was to evaluate how cricketers were coached to bat since junior cricket.

## Methodology

A mixed-methods research study in which an online survey (using both open and closed-ended questions) was distributed to cricket players (n = 100) from South Africa. The online survey was adapted for use through a previously validated questionnaire used among cricket coaches. Pearson’s Chi-squared tests were used to determine if there were any significant trends in the answers from the survey. The results of the survey were then coded and transferred to SPSS (Statistical Software, IBM, Version 27). The level of significance was set at p<0.05.

## Results

This study showed that most cricket players (76%) were shown how to lift their bat while batting by their first cricket coach. It was also discovered that 63% of players were taught to direct their bat towards the off-side instead of towards the wicket-keeper or the stumps, while 43% of players (χ2 = 17.54, df = 6, p = 0.008) were taught to have an open face when directing their bat. It was revealed that in most cases (42%; χ2 = 11.65, df = 6, p = 0.7), the player adapts their technique, while 27% of participants do not adapt their technique for the different formats of cricket.

## Conclusion

Recommendations from this study include the emphasis on the lateral batting backlift technique (LBBT) as it has been shown to be the more successful backlift and a prominent characteristic of cricket batters at the highest level. Despite a number of available coaching aids to assist in the training of junior cricketers, players should be allowed to express themselves individually. Further research (and a policy) is required to compile a coaching framework to teach other elements of the batting technique to assist in cricket batting coaching.

# Abstract: E3: QUANTIFYING COACHING CONSIDERATIONS, ATTITUDES AND STRATEGIES TO PLAYER SUBSTITUTIONS IN SCHOOL RUGBY UNION

**Published:** 2022-01-01

**Authors:** Cheryl-Ann Volkwyn, Andrew Green, Rian Lombard

**Affiliations:** 1 University of Johannesburg

## Background

Rugby is a high-intensity, intermittent collision sport played by fifteen players on each team and requires highly specialised playing positions. Due to these physical demands and to maintain a highly competitive level, coaches may substitute no more than eight players during the match. Player substitutions can affect the match’s outcome and, knowing when to substitute players, is largely determined by a coach’s intuition. Therefore, the coach’s involvement and decisions behind player changes must be assessed, as the effects of substituting a player can be either positive or detrimental to the team’s performance.

## Methodology

A six-sectioned questionnaire was developed in conjunction with five university and professional coaches and aimed to assess various issues surrounding player substitutions. The six sections included demographics, reasons for substituting players, considerations, informing players, match progression and status. Question responses followed a five-point Likert scale. A total of sixty-nine age-group level rugby coaches (experience 11.7 ± 9.0 years) completed the questionnaire.

## Results

Common responses indicated that coaches used substitutions to increase their teams’ chances of winning and to reduce player load. Additionally, the results indicate that coaches are likely to substitute players based on a predetermined strategy and to manage player loads. Coaches were likely to change players following a team scoring and as the game progressed. Additionally, substitutions were more likely when game importance increased. Responses indicated that coaches frequently inform players with adequate time prior to substitutions. The most frequent time of communication occurred during half-time. Finally, coaches frequently considered the technical abilities of the players, timing and score of the game before changing players.

## Conclusion

The results provide a likely indication that coaches rely on their own previous playing experience regarding their decision-making approaches. As this data were collected from school-level rugby teams, it is likely that coaches afforded player game time closer to the completion of a winning match. Importantly, substitutions are planned and not reactive. Overall, coaches should provide sufficient notice and instruction to replacement players. Improved substitution timing could occur when coaches are made aware of and respond to visual clues of players’ performances to enforce substitutions.

# Abstract: E4: EPIDEMIOLOGY OF UPPER LIMB INJURIES IN THE SUPER RUGBY TOURNAMENT (2013–2016)

**Published:** 2022-01-01

**Authors:** Tshegofatso Gaetsewe, Audrey Jansen van Rensburg, Martin P. Schwellnus, Christa Janse van Rensburg, Charl Janse van Rensburg, Esme Jordaan

**Affiliations:** 1 Section Sports Medicine, Faculty of Health Sciences, University of Pretoria, Pretoria, South Africa; 2 Sport, Exercise Medicine and Lifestyle Institute (SEMLI), Faculty of Health Sciences, University of Pretoria, Pretoria, South Africa; 3 Emeritus Professor of Sport and Exercise Medicine, Faculty of Health Sciences, University of Cape Town, South Africa; 4 International Olympic Committee (IOC) Research Centre, South Africa; 5 Biostatistics Unit, Medical Research Council, South Africa; 6 Statistics and Population Studies Department, University of the Western Cape, South Africa

## Background

Rugby is a contact and high-impact collisions sporting code, with a high risk of causing acute or long-term injury. This study aimed to determine the incidence and nature of upper limb injuries sustained by elite rugby players during the annual Super Rugby tournaments (2013 to 2016).

## Methodology

This study was a cross-sectional analysis of data collected prospectively among 868 rugby players from six South African teams who participated in the annual Super Rugby tournament. Team physicians collected daily injury data including the size of the squad, the type of day, player position, whether it was a training or match injury, hours of play (training and matches). Other data collected include body area, tissue type, severity (days lost), mechanism of injury (contact vs. non-contact), and phase of play. The incidence (per 1000 player days; 95% CI) and illness burden (IB: days lost due to injury per 1000 player-days) are also reported

## Results

A total of 776 rugby-related injuries were recorded during the study period, of which upper limb injuries accounted for 20.8% of all rugby injuries with an incidence of 2.1 per 1000 player days (95% CI: 1.8–2.5); 2). The incidence of upper limb injuries sustained during match-play was 23.3 per 1000 player days (95% CI: 19.8–27.3), forwards incurred 57.2% and backs 42.8% upper limb injuries during match-play; the shoulder/clavicle (52.8%) was the most injured locations; muscle/tendon (44.1%) and joint/ligament (42.1%) account for the majority of injuries. Contact events (96.1%) were the main cause with an incidence of 22.4 per 1000 player days (95% CI: 18.4–26.3); in particular, during a tackle (46.7%). More than 60% of shoulder injuries resulted in minimal to mild (2–7 days) time-loss.

## Conclusion

Upper limb injuries accounted for 20.8% of all rugby injuries. The shoulder/clavicle is the most frequent region injured, muscle/tendon and joint/ligament were the common tissue type. Most players sustained minimal to moderate severity during match-play and contact events were the main cause of upper limb injuries. Injury surveillance data inform sports physicians and coaches about keeping players safe from injury, implementation of prevention strategies and optimal management of injuries will ensure the quickest and safest return to play following injury.

# Abstract: F1: EMPLOYEE’S PERCEPTIONS OF LEISURE AND RETIREMENT DURING THE TWELVE MONTHS BEFORE RETIREMENT: A DESCRIPTIVE INTERPRETIVE STUDY IN THE KENNETH KAUNDA DISTRICT OF THE NORTH-WEST PROVINCE

**Published:** 2022-01-01

**Authors:** Lulama Mabala, Theron Weilbach

**Affiliations:** 1 NWU PhASRec

## Background

The adjustment and adaptation process to retirement can be challenging and stressful for retirees. Leisure can aid in the retirement adjustment process by providing the structure that is lost during retirement and provide benefits such as physical, cognitive, psychological, social, and self-developmental benefits. Planning for leisure is an essential component of planning for the retirement adjustment which is frequently overlooked by pre-retirees. A potential lack of adequate leisure planning before retirement can impact a person’s overall health during retirement. This study aims to describe and interpret perceptions of leisure and retirement during the 12 months before the retirement of blue and white-collar employees in the Kenneth Kaunda District. The dynamic theory of resources forms the theoretical foundation of the study. This approach provides a theoretical framework in which an understanding, anticipation, and examination of the relationships between people’s resources and their adaptation to retirement will be created.

## Methodology

The participants will include at least 12 white- and 12 blue-collar workers in the year before retirement. To gather data semi-structured face-to-face audio-recorded interviews will be conducted. Data will be analysed by firstly transcribing the audio-recorded interviews, then a microscopic examination will be conducted to bring out the detailed, intensive, complexity of the data. Once the microscopic examination is completed, the data will be analysed through a thematic process by using ATLAS.ti, which is a powerful workbench for qualitative data analysis. Lastly, for quality purposes, a co-coder will be used for coding.

## Results

N/A

## Conclusion

To date, Covid-19 posed a significant challenge. Ultimately, it is expected that this study will provide valuable information to pre-retirees to make better-informed decisions to optimally adapt to retirement life. Not only will it benefit the targeted study population but will also benefit organisations in the public and private sectors by guiding and showing them what should be included in a retirement plan. Additionally, individuals who are proactively planning for their retirement may realise the importance of leisure before their actual retirement and make appropriate changes to their leisure behaviour.

# Abstract: F2: PATTERNS OF PARTICIPATION IN CAMPUS RECREATION SPORT AND LEISURE BOREDOM

**Published:** 2022-01-01

**Authors:** Cornelia Schreck, Nadine Labuschagne, Cornelia M. Schreck, Theron Weilbach

**Affiliations:** 1 PhASRec, North-West University

## Background

Undergraduate students can experience leisure boredom when they are not exposed to Campus Recreation Sport (CRS), which in turn can lead to feeling unhappy, developing low self-confidence, abusing substances, and in the end dropping out of university. More than one quarter (28.5%) of students in South Africa (SA) drop out of university at the end of their first year after entering a four-year degree programme. A solution to students dropping out of university might be to increase student involvement and participation in campus recreation. The purpose was to determine the participation patterns in CRS of undergraduate students at a South African university and to what degree they experience leisure boredom.

## Methodology

A once-off cross-sectional design was used. The sample consisted of 581 students. An online survey consisting of various research instruments, including a demographic questionnaire, frequency and format of participation in recreational sport questionnaire, and items related to leisure boredom from the leisure experience battery for young adults by Barnett (2005) was used.

## Results

There were statistically significant differences between the gender groups’ frequencies of taking part in netball (p=0.010) and social dancing (p=0.044). There were statistically significant differences between all racial groups’ leisure boredom (p=0.000). Medium to large practical significant differences was found between other/coloured (d=0.9), coloured/white (d=0.7) and other students and African students (d=0.6). Statistically significant differences (p=0.017) for leisure boredom in the total sample regarding all three different accommodation types were found.

## Conclusion

Most students prefer to participate on their own or with their friends sharing the same interests, but not all on-campus activities cater to individual participation. This could be that students are more likely to choose activities that provide social opportunities with the social factor as the second-highest-rated motivational factor for CRS participation. Only registered undergraduate students from one university participated in the survey, thus limiting the researcher with comparisons.

# Abstract: G1: CARDIOPULMONARY EXERCISE TESTING COMBINED WITH CARDIAC REHABILITATION EXPOSING ADDITIONAL ARRHYTHMIAS WITHIN AEROBIC METABOLISM, POTENTIALLY DUE TO OXIDATIVE STRESS – IS THIS THE MISSING LINK IN UNEXPLAINED CARDIOVASCULAR DEATHS?

**Published:** 2022-01-01

**Authors:** Johann Els

**Affiliations:** 1 HPCSA

## History

“Cardiac arrhythmia occurs frequently worldwide, and in severe cases can be fatal. Mitochondria are the power plants of cardiomyocytes. In recent studies, mitochondria under certain stimuli produced excessive reactive oxygen species (ROS), which affect the normal function of cardiomyocytes through ion channels and related proteins. Mitochondrial oxidative stress (MOS) plays a key role in diseases with multifactorial etiopathogenesis, such as arrhythmia; MOS can lead to arrhythmias such as atrial fibrillation and ventricular tachycardia.” – Liu et al, 2022

## Physical Findings

Additional arrhythmias and premature ventricular contractions are exposed within the aerobic metabolism through ECG monitoring, which the anaerobic stress ECG cannot expose - the aerobic ECG can potentially clarify unexplained cardiovascular disease. Literature suggests that “electrical instability and electrical remodelling underlying the arrhythmia may result from a cellular energy deficit and oxidative stress, which are caused by mitochondrial dysfunction.” Clinical and sub-clinical data are provided. These observations can only be exposed through an in-depth analysis of various physiological variables following a resting metabolic rate assessment and cardiopulmonary exercise testing. Individuals that were monitored for an extended period showed additional clinical pathology (that would not have been exposed previously) during physical exercise. This potentially showed weakened physiological systems that could expose oxidative stress, which might play an underlying role in unexplained pathology of various chronic diseases.

## Differential Diagnosis/Hypothesis

The clinical importance of ideal oxygen supply and uptake on a cellular level (during exercise) is not yet fully understood. Once the weakest cell is exposed to the optimal oxygen supply or uptake, the true functional status of the cell seems to produce oxidative stress (due to mitochondrial dysfunction), causing the true underlying clinical status to be exposed, i.e., electrical instability within the heart. The sinus rhythm appears to be normal, leading to a hypothesis that the clinical pathology present is within the mitochondria of the heart - in need of rehabilitation. Exercise within the aerobic metabolism releases oxidative stress, preventing further damage to various intracellular substances that affect normal functioning of the body, and cause of disease.

## Test and Results

Subjects underwent the following battery of tests:

Components of Screening and risk profiling on the Medicise Patient Management System consist of:

1. Completing the PAR-Q questionnaire
2. Informed consent.
3. Exercise participation
4. Known diagnoses (cardiovascular/metabolic/renal)
5. Signs and symptoms suggestive of cardiovascular/metabolic/renal disease
6. Risk factors
7. Medical history

Cardiometabolic assessments include:

- Resting metabolic rate assessment
- Spirometry
- Cardiopulmonary Exercise Test

The sample consisted of 14 men and women (after referral for exercise training with non-communicable diseases); age distribution: 17 – 81 years, both male and female. Risk profiling was done through screening and pre-exercise evaluation protocol as per guidelines by the American College of Sports Medicine. Data from a resting metabolic rate towards maximal exertion were gathered using MetaLyzer^®^3B and MetaSoft^®^Studio software (analysing 86 physiological variables/calculations) from CORTEX Biophysik GmbH and Custo Cardio 300 (analysing comprehensive ECG variables) from Custo Med GmbH. A physiological analysis was followed to identify the ideal exercise intensity according to the optimal oxygen supply and uptake zone. The results showed the clinical importance of cardiopulmonary exercise testing in the sample group. Internationally “cardiopulmonary exercise testing applications in cardiology have grown impressively to include all forms of exercise intolerance.” A leading cardiology practice in the Western Cape provided the following interpretation of the 14 case studies - “From the data that you showed - my biggest clinical concern is that some of your patients may have a very high load of Ventricular Ectopics and by implication, some of your data points even suggest that certain patients may be having runs of ventricular tachycardias-which is very dangerous if true. There are also suggestions in your data that there may be other arrhythmias which are elicited during exercise such as focal atrial tachycardias etc.”

## Final/Working Diagnosis

Each individual either has a functional or clinical physiological weakness within integrative physiology. Identifying the physiological cause of exercise intolerance is the essence of understanding the clinical problem. Once this weakness is exposed to the optimal oxygen supply or uptake quantity within the integrative physiology, the human body will be provided with the chance to initiate the healing process through oxygen exposure.

## Treatment and Outcomes

All subjects participated in exercise using Custo Med Diagnostic Standard Cardiac Rehabilitation Software to monitor cardiovascular response during exercise with ECG monitoring. Subjects were monitored for a minimum of three (3) and a maximum of 259 exercise sessions (minimum 20 minutes at a time) while ECG monitoring provided clear information of the cardiovascular response during exercise. ECG monitoring allowed for a comprehensive, and correct profile of arrhythmia and premature ventricular contractions during exercise within the aerobic metabolism. Individuals that were monitored for an extended period showed clinical pathology during exercise for clinical interpretation during physical exercise. This led to a better understanding of the specific physiological systems that needed prolonged exposure to optimal oxygen. Important to note that most subjects could reduce cardiac arrhythmias while increasing the first anaerobic threshold and peak oxygen uptake significantly through participation in cardiac rehabilitation with ECG monitoring.

## Clinical Case Study

78-year-old active male with the following medical history:

- Coronary artery disease
- Concomitant respiratory disease
- Covid-19 infection (March 2020)
- Hypertension
- Diabetes
- Gout

Results of 259 exercise sessions over a period of 16 months with ECG monitoring:[Fig f1-2078-516x-34-v34i1a14886]

**Figure f1-2078-516x-34-v34i1a14886:**

**Table:** 

First Anaerobic Threshold before 259 exercise sessions	First Anaerobic Threshold after 259 exercise sessions
**1.04 L**	**1.62 L**

**Table:** 

Peak Oxygen Uptake before 259 exercise sessions	Peak Oxygen Uptake after 259 exercise sessions
**1.44 L**	**2.21 L**

# Abstract: G2: BERTOLOTTI’S SYNDROME: OVERLOOKED, UNDER-DIAGNOSED?

**Published:** 2022-01-01

**Authors:** Kobus Slabber

**Affiliations:** 1 SASMA

## History

A comparative presentation about two cases of Bartolotti’s Syndrome - one treated conservatively and one that needed surgical intervention.

### Case A

24 y/o Female with two-month history of gluteal/lower back pain, spreading down her leg when sitting and sprinting. Unresponsive to physiotherapy and anti-inflammatory treatments.

### Case B

45 y/o Female with lower back pain, claudication type pain spasms that did not improve on physiotherapy and anti-inflammatory treatments.

## Physical Findings

### Case A

Tender with slightly palpable protrusion over the piriformis area. Normal general and neurological evaluation.

### Case B

Tender over L-spine, no other musculoskeletal abnormalities. Normal general and neurological evaluation.

## Differential Diagnosis/Hypothesis

### Case A

Sciatica, Piriformis syndrome, SI-joint/facet arthropathy, Lumbar radiculopathy - annular tear/discus protrusion.

### Case B

Lumbar arthropathy/radiculopathy - foraminal stenosis/discus protrusion

## Test and Results

### Case A

Ultrasound (Hamstring calcification), X-rays (sacralisation), CT-scan & MRI to be done under consent for academic reasons of submission accepted.

### Case B

X-rays (sacralisation), CT-Scan (bilateral sacralization), MRI (Left L5 nerve compression).

## Final/Working Diagnosis

### Case A

Bertolotti’s Syndrome with right-sided L5 radiculopathy.

### Case B

Bertolotti’s Syndrome with left-sided L5 radiculopathy.

## Treatment and Outcomes

### Case A

Cortisone and local infiltration - pain-free.

### Case B

Cortisone and local infiltration - unsuccessful. Anterior Sacrolumbar decompression - pain-free.

# Abstract: H1: A COMPARISON OF THE RISK PROFILE FOR DEVELOPING ILLNESS-RELATED MEDICAL ENCOUNTERS IN HALF- AND ULTRAMARATHON RUNNERS

**Published:** 2022-01-01

**Authors:** DC Baker, PS Wood, TCdS Camacho, MP Schwellnus, S Swanevelder, E Jordaan

**Affiliations:** 1 Division of Biokinetics and Sports Science, Department of Physiology, Faculty of Health Sciences, University of Pretoria, South Africa; 2 Sport, Exercise Medicine and Lifestyle Institute (SEMLI), Faculty of Health Sciences, University of Pretoria, South Africa; 3 International Olympic Committee (IOC) Research Centre, South Africa; 4 Emeritus Professor, Faculty of Health Sciences, University of Cape Town, South Africa; 5 Biostatistics Unit, South African Medical Research Council; 6 Biostatistics Unit, South African Medical Research Council; Statistics and Population Studies Department, University of the Western Cape

## Background

Research comparing risk profiles for medical encounters in race entrants at distance running events is limited. The aim of this study was to determine and compare the risk profile for developing illness-related medical encounters in half-marathon compared to ultramarathon runners.

## Methodology

Online pre-race medical screening questionnaire data from 76654 consenting race entrants (71.8% of all entrants) over four years of Two Ocean Marathon (2012–2015) were analysed, using a prospective cross-sectional observational study design. Study participants were classified into four risk categories (‘very high risk’, ‘high risk’, ‘intermediate risk’ and ‘low risk’) based on the history of the following: existing cardiovascular disease (CVD), history of any symptoms of CVD, or any risk factor for CVD, disease in other organ systems, medication use and history of collapse in half-marathon and ultramarathon. We report the prevalence (%; 95%CI) in each risk category for half- and ultra-marathon entrants.

## Results

Most entrants in the half-marathon race were classified in the “low” risk category (43.20%; 95% CI 42.7–43.7), followed by “intermediate” (41%; 95% CI 40.5–41.6) and “high” (12.6%; 95% CI 12.2–13.0) risk categories. The majority of entrants in the ultramarathon were classified in the “intermediate” (51.90%; 95% CI 51.2–52.6) risk category, followed by “low” (36.20%; 95% CI 35.5–36.9) and “high” (9.10%; 95% CI 8.7–9.4) risk categories. Additional findings include higher prevalence of an existing CVD in half-marathon runners (1.66%; CI 1.53–1.81) compared to ultramarathon runners (1.36%; 95% CI 1.21–1.54), and a higher prevalence of risk factors for CVD in half-marathon runners (12.46%; CI 12.11–12.81) compared to ultramarathon runners (9.27%; 95% CI 8.89–9.67).

## Conclusion

Half-marathon runners had a higher prevalence of existing CVD and CVD risk factors than ultramarathon runners. However, more than half of the ultramarathon runners reported (a) existing chronic disease in other organs, (b) use of prescription medication, (c) use of anti-inflammatory drugs, and (d) history of collapse during exercise.

# Abstract: H2: EYE PATHOLOGIES IN PARA ATHLETES IN THE WINTER SETTINGS: AN ANALYSIS OF THE SOCHI 2014 AND PYEONGCHANG 2018 PARALYMPIC WINTER GAMES

**Published:** 2022-01-01

**Authors:** Lovemore Kunorozva, Ali Ganai, Phoebe Runciman, Wayne Derman

**Affiliations:** 1 Institute of Sport and Exercise Medicine (ISEM), Division of Orthopaedic Surgery, Faculty of Medicine and Health Sciences, Stellenbosch University, Stellenbosch, South Africa

## Background

Eye pathologies have a negative effect on a Para athlete’s performance and well-being. An in-depth description of the nature and presentation of these eye pathologies could assist in developing prevention strategies for such pathologies in future. The aim of this study was to describe the nature of eye pathologies presenting in Para athletes during the Sochi 2014 and PyeongChang 2018 Paralympic Winter Games.

## Methodology

This study investigated eye pathologies in Para athletes using the Web-Based Injury and Illness Surveillance (WEB-IISS) tool during the 2014 Sochi and 2018 PyeongChang Paralympic Winter Games. Descriptive data were obtained and analysed from a de-linked database containing Para athlete information on eye illnesses and injuries from the two Winter Paralympic Games.

## Results

Ice sledge hockey (30.3%), nordic (19.2%) and alpine (18.4%) skiing, accounted for the sports with most of the eye pathologies reported during the two Winter Games. Furthermore, the majority of eye pathologies were reported in athletes with an amputation or lower limb deficiency (66.9%), visual impairment (18.4%) and those with a spinal injury (11.9%).

## Conclusion

Male Para athletes, mostly taking part in ice sledge hockey and those with lower limb deficiency or visual impairment suffer more eye pathologies during Winter Paralympic Games. Furthermore, environmental eye illnesses and allergic conjunctivitis account for just below half of all eye illnesses and were associated with snow glare/dry eye and pre-existing allergy or asthma conditions, respectively. The findings from this study may aid the development of eye pathology prevention strategies in Winter sport settings.

# Abstract: H3: EYE PATHOLOGIES IN PARA ATHLETES IN THE WINTER SETTING- A SYSTEMATIC REVIEW

**Published:** 2022-01-01

**Authors:** Lovemore Kunorozva, Ali Ganai, Phoebe Runciman, Wayne Derman

**Affiliations:** 1 Institute of Sport and Exercise Medicine (ISEM), Division of Orthopaedic Surgery, Faculty of Medicine and Health Sciences, Stellenbosch University, Stellenbosch, South Africa

## Background

Eye illnesses can be detrimental to athletes’ performance and well-being. These ocular illnesses may be an inherent finding in athletes with visual impairment, however acute eye illness might also be present in athletes with other impairments. Yet, these illnesses have not been explored in Para-athletes. The aim of this systematic review was to describe the epidemiology of eye pathology presenting in Para athletes in both Winter and Summer sports settings.

## Methodology

A comprehensive literature search of eye pathologies presenting in Para athletes was conducted employing PubMed, Ebscohost and Web of Science databases up to June 2021. Each of the articles was independently reviewed for relevance and inclusionary criteria, with nine studies meeting these criteria.

## Results

Overall, the quality of the included studies was excellent. Illnesses in the eye and adnexa were more prevalent in Winter (IR: 1.6 – 2.2/1000 athlete days) compared to Summer (IR: 0.3–0.5/1000 athlete days) sports settings. Eye pathologies were reported in athletes with limb deficiencies (22.4%), spinal cord injury (20.3%), visual impairment (15.1%) and central neurology injury (14.8

## Conclusion

The findings of this review indicate that: 1) Eye pathology in Para athletes is an understudied area; 2) Eye pathology is present in athletes other than those with visual impairment. There is a need for further research on eye pathology, particularly in Winter sports settings in order to understand the types and nature of eye pathologies that affect Para athletes during competition and training settings

# Abstract: H4: AN ANALYSIS OF THE PARTIAL FEASIBILITY OF A NOVEL CARDIAC EXERCISE REHABILITATION PROGRAMME FOR PATIENTS SUFFERING FROM CARDIOVASCULAR DISEASE

**Published:** 2022-01-01

**Authors:** Tayla Ross, Jacolen Kroff, Elizm Atterbury

**Affiliations:** 1 University of Cape Town

## Background

South Africans are facing a growing problem of cardiovascular disease (CVD), which has major implications for healthcare services and has placed increasing strain on the struggling South African healthcare system. Cost-effective prevention and management measures are needed to slow down the growing CVD epidemic and relieve strain on healthcare systems. The need exists for more evidence to demonstrate that cardiac exercise rehabilitation programmes (CRPs) can significantly reduce readmissions, mortality, comorbidities, and improve the quality of life throughout the country. The aims of this study were to determine the partial feasibility of a novel CRP in a South African public hospital setting to provide optimal and sustainable healthcare to CVD patients.

## Methodology

Partial feasibility was assessed by evaluating the recruitment potential and sample population characteristics of the target population, through the retrospective analysis of the Victoria Hospital VIMRI database, and by evaluating the test-retest reliability of the prospective protocol testing and monitoring measures through 3 different methods.

## Results

209 participants were assessed according to inclusion and exclusion criteria. 109 (52.2%) were considered ineligible for a CRP, whereas 100 individuals (47.8%) were considered eligible. There were no significant differences between the populations in terms of anthropometry, however, there were significant differences for 4 comorbidities and 2 medications. 22 outcome measures were assessed for reliability, 5 were classed as having “poor” reliability, 9 as “moderate”, 3 as “good” and 5 as “excellent’. 18 measures had a lower than small effect size (d<0.2), indicating near identical repeated measures and excellent test-retest reliability. The remaining 8 had small effect sizes between 0.2–0.5.

## Conclusion

It is expected that 33 patients (100 participants/3 months) will be eligible for the CRP every month, which is nearly triple the expected intake, exceeds prospective resource and staff capacities and is greater than what can currently be accommodated. We recommend that the number of resources and trained staff be increased before CRP implementation. The majority of the monitoring tools assessed had acceptable test-retest reliability. However, the methods used to measure blood pressure, oxygen saturation and rate of perceived exertion were questionable. We recommend that alternative devices are utilised for these variables.

# Abstract: H5: A SCOPING REVIEW EXAMINING PHYSICAL ACTIVITY INTERVENTIONS FOR THE MANAGEMENT OF HYPERTENSION

**Published:** 2022-01-01

**Authors:** Mandisa Simamane

**Affiliations:** 1 University of the Witwatersrand

## Background

As populations age, adopt more sedentary lifestyles, and increase their body weight, the prevalence of hypertension worldwide is continuing to rise. One of the main non-pharmacological strategies for prevention, control, and treatment of hypertension and its future complications is physical activity. Aim: The main aim of the study was to identify the type of physical activity intervention that can assist hypertensive patients in managing hypertension. A Scoping review was used as a drive toward evidence-based practices and as a technique to map relevant literature and the types of evidence available with regards to management of hypertension using physical activity.

## Methodology

A systematic computer-based search was undertaken through the University of the Witwatersrand/LibGuides/Electronic Resources/Database A-Z. Search words were as our inclusion criteria of (1) hypertension/high blood pressure, (2) exercise/physical activity, (3) intervention/training intervals, (4) frequency, intensity, time & type, (5) for the period of 2009 to 2020.

## Results

The results led to 32 randomised controlled/experimental studies which fulfilled the criteria required and included: what study design was used, where was the study located, what was the sample size, and what sex and race/ethnicity. A 16-week lifestyle intervention significantly improved cardiovascular risk factors in all groups, and pharmacological therapy was largely reduced or removed completely.

## Conclusion

Concluding, it was found that different exercise modalities elicit very different changes in blood pressure variability while they induce similar reductions in blood pressure values. Caution and good judgment should be used when prescribing more rigorous exercise for those with high blood pressure. The data showed that elderly hypertensive individuals undergoing pharmacological treatment may benefit from practising resistance exercise and that their pressure will probably not go up as a result. These data suggest that antihypertensive treatment can also reduce the concern that physical exercise can produce an extreme and acute hypertension response in hypertension subjects. The use of aerobic exercise is a gold standard exercise to reduce blood pressure. even for patients who have well-controlled BP, aerobic exercise performed alone is an effective strategy to reduce BP during the hours after the cessation of exercise.

# Abstract: I1: MOTIVATION FOR EXERCISE AND THE EFFECTS OF EXERCISE PARTICIPATION ON MENTAL HEALTH DURING THE COVID-19 LOCKDOWN IN SOUTH AFRICA

**Published:** 2022-01-01

**Authors:** Courtenay M. Davis, Heather Morris-Eyton

**Affiliations:** 1 Department of Sport and Movement Studies, University of Johannesburg

## Background

The coronavirus (COVID-19) pandemic posed a significant threat to the physical and mental health of the South African population. It changed the way in which individuals were able to exercise in order to maintain physical fitness during the various levels of government enforced lockdown restrictions. The purpose of this study was to draw a comparison of the levels of motivation between virtual and non-virtual workouts which occurred during the COVID-19 lockdown in South Africa.

## Methodology

An internet-based survey examined in part exercise motivation between virtual and non-virtual workouts during the lockdown. This was an adapted version of the SRQ-E as it explored the reasons for exercising and whether participants’ motivation for exercising was intrinsic or extrinsic. Participants (n=120) were residing in South Africa and had to have completed at least one virtual and non-virtual workout during the COVID-19 lockdown (March 2020 – February 2021). A descriptive and comparative analysis was conducted to gain insight into the comparison between the motivation of the different workout regimens, and why participants preferred their workout regimen of choice.

## Results

During the lockdown, virtual workouts were enjoyed most often (52%). This was primarily due to the social setting (32%) where participants felt they were still engaging in exercise with others, and the interaction with their instructor (32%). Participants who preferred non-virtual home-based workouts reported that they could work out at a time convenient to them (68%) and at their own pace and fitness level. Participants who favoured non-virtual workouts were intrinsically motivated to participate in exercise programmes (M=5,92, SD=0,91,) when compared to those who preferred virtual workouts (M=5,48, SD= 1,10). Men (M=5,99, SD= 0,90) were more intrinsically motivated to engage in workouts during lockdown than women (M=5,52, SD= 1,07). The participation in physical activity during the Covid-19 lockdown played a role in boosting mental health as it positively increased 92% of the participants’ mood

## Conclusion

Physical activity had a positive effect on mental health during the lockdown. Participating in virtual workouts was the preferred mode of exercise, however, those who partook in non-virtual workouts showed higher levels of intrinsic motivation.

# Abstract: I2: PHYSIOLOGICAL RISK PROFILES OF ASYMPTOMATIC MALES PERFORMING THE SALAAH (ISLAMIC PRAYER) AS A LOW-INTENSITY PHYSICAL ACTIVITY

**Published:** 2022-01-01

**Authors:** Abdul Hamid Jalal, Habib Noorbhai

**Affiliations:** 1 Department of Sport and Movement Studies, Faculty of Health Sciences, University of Johannesburg, South Africa

## Background

The Islamic Prayer (Salaah) has been found to be a type of low-to-moderate intensity aerobic exercise and has physiological and psychological benefits for one’s health. The aim of this study was to investigate and determine the physiological risk profiles using selected clinical measurement tools.

## Methodology

Using a cross-sectional research design, an intervention study of selected morphological, cardiovascular and neuromuscular variables on a sample of asymptomatic males (n = 20), aged 21 – 40 years, was conducted. Resting heart rate (RHR), resting systolic blood pressure (SBP), resting diastolic blood pressure (DBP), body mass index (BMI) and percentage body fat (BF%) were measured prior to a Salaah simulation activity. Electromyography (EMG) measurements were performed on the vastus medialis oblique (VMO) for a single maximum voluntary contraction (MVC) along with two movement transitions of the Salaah. Bilateral manual muscle strength testing (MMT) using a myometer was conducted on the quadriceps femoris muscle group. Using SPSS (Version 27, IBM), descriptive and inferential statistical analyses (including Pearson’s correlation coefficient to explore links between all variables in the study) were conducted. The level of significance for these correlations was set at p<0.05.

## Results

Negative correlations were found between RHR and MVC average (r = −0.03; p = 0.91) ; post Salaah SBP and MVC average (r = 0.19; p = 0.42) ; Post Salaah DBP and the MVC average (r = −0.41; p = 0.08). Positive correlations were found between the MVC average with right (r = 0.14; p = 0.56) and left (r = 0.18; p = 0.44) quadriceps femoris MMT and between BMI and BF% (r = 0.69; p = 0.00). RHR (r = 0.26; p = 0.27), resting SBP (r = 0.49; p = 0.03), post Salaah SBP (r = 0.27; p = 0.26) and post Salaah DBP (r = 0.36; p = 0.12) were positively correlated with BMI.

## Conclusion

This study demonstrated that a sample of asymptomatic males who perform the daily Salaah were within reasonably healthy norms. The study also demonstrated that a physiological benefit can exist by the Salaah as a form of light-intensity aerobic activity in clinical exercise rehabilitation.

# Abstract: I3: BARRIERS TO PARTICIPATION IN SPORT AMONG URBAN ADOLESCENTS ATTENDING AN INTERNATIONAL SCHOOL IN CAPE TOWN, SOUTH AFRICA

**Published:** 2022-01-01

**Authors:** Robyn Klaasen, Maya van Gent, Habib Noorbhai

**Affiliations:** 1 University of Fort Hare; 2 University of Johannesburg

## Background

The global decline in participation in physical activity and sport by adolescents remains a cause for concern. Few of the many studies that have been conducted have concerned South Africa, specifically outside of a rural or disadvantaged context. The purpose of this study was to identify the principal barriers to participation in sport and make a comparative appraisal of their influence on male and female adolescents at an international school in Cape Town, South Africa.

## Methodology

The Barriers to Sport Participation Questionnaire (BSPQ) was completed by 107 voluntary respondents. The data was validated by a Kaiser-Meyer-Olkin (KMO) value of 0.808 and a Chi-square value of 2403.664 (p = 0.000; df = 820).

## Results

The results revealed seven principal barriers, of which fear of academic failure, environmental constraints, and lack of interest were the most influential. It was also established that the barriers exerted the most influence on female respondents and on those respondents who either did not participate in sport or were from households whose other members did not do so.

## Conclusion

The findings underscored the need for education and exposure to the sport and the need to promote the sport in response to specific disparities, even if doing so entails the implementation of segregated programmes.

# Abstract: I4: THE RELATIONSHIP BETWEEN BARRIERS TO PARTICIPATION IN SPORT FOR ADOLESCENT LEARNERS AND THE GRADE (SCHOLASTIC YEAR) IN WHICH THEY ARE ENROLLED: A CASE STUDY IN AN INTERNATIONAL SCHOOL IN CAPE TOWN, SOUTH AFRICA

**Published:** 2022-01-01

**Authors:** Robyn Klaasen, Maya van Gent, Habib Noorbhai

**Affiliations:** 1 University of Fort Hare; 2 University of Johannesburg

## Background

Participation in sport among adolescents continues to decline at an alarming rate throughout the world. While several studies have been conducted to identify barriers to participation, limited research, particularly in South Africa, has been conducted to detect correlations between specific barriers and other variables. The purpose of this study was to identify barriers to participation in sport among urban adolescents and determine correlations between individual barriers and grade (scholastic year) in an international school in Cape Town, South Africa.

## Methodology

The Barriers to Sport Participation Questionnaire (BSPQ) was completed by 107 learners at the school. The data was validated by a Kaiser-Meyer-Olkin (KMO) value of 0.808 and a Chi-square value of 2403.664 (p = 0.000; df = 820).

## Results

Seven principal barriers were identified, of which fear of academic failure, environmental constraints, and lack of interest were the most influential. Regression analysis revealed that the fear of academic failure, health and injury-related concerns, and no relevance or priority barriers were the most significant, particularly among Grade 7 and Grade 9 learners.

## Conclusion

Consequently, it appears probable that barriers to participation in sport could be specific to particular school year and that interventions need to be specific, appropriate, well-timed, and effectively implemented during the middle school years of Grades 6 to 8, to encourage sustained engagement with sport and mitigate the global decline.

# Abstract: J1: RELATIONSHIPS BETWEEN GROUND REACTION FORCE, ISOKINETIC KNEE STRENGTH, AND THE INCIDENCE OF LOWER EXTREMITY INJURIES IN UNIVERSITY-LEVEL NETBALL PLAYERS

**Published:** 2022-01-01

**Authors:** Henriette Hammill, Lenthea Kamffer, Mark Kramer, Yolandi Willemse

**Affiliations:** 1 Physical Activity, Sport, and Recreation (PhASRec) Research Focus Area, North-West University, Potchefstroom, South Africa; 3 Center for Health and Human Performance, North-West University, Potchefstroom, South Africa

## Background

Given the physical demands (e.g., agility), outcomes (e.g., potential injury), and increasing competitiveness of netball, the monitoring of certain performance characteristics has become increasingly important in order to better understand the injury risk factors at play. The repeated high-impact loading on the lower extremity joints increases the risk of injury. Knee strength provided during the co-contraction of the hamstring- and quadriceps muscle groups give dynamic stabilization which is particularly important during landing. The aim of this study is to perform a literature review on the incidence of lower extremity injuries in netball players.

## Methodology

A typical PRISMA literature surveillance is applied: An electronic literature search was conducted using the key search terms “netball”, “ground reaction force”, “isokinetic knee strength”, “lower extremity injuries”, “landing kinetics”, “time to stabilisation”, “force plate”, “dynamometer”, “eccentric”, “injury mechanics”. Peer-reviewed, English-written research articles were included in this literature review.

## Results

There is a high incidence (>50%) of lower extremity injuries in netball players.

## Conclusion

The fact that netball is a fast-paced, multi-directional movement sport with many jumps, leaps and landings, the resultant GRF is a significant risk factor to consider when investigating lower extremity injuries. Knee muscular strength contributes to the stability of the lower extremities during landing from a jump and along with GRF, isokinetic knee strength plays a vital role when investigating lower extremity injuries.

This literature review regarding the relationship between GRF, isokinetic knee strength and the incidence of lower extremity injuries in university-level netball players will aid valuable information towards conditioning protocols, training loads, rehabilitation- and prehabilitation protocols, lowering the risk for injuries, as well as re-occurrence of previous injuries and optimising performance.

# Abstract: J2: KINEMATIC GAIT COMPARISON BETWEEN BACK-CARRIED-, AND NON-BACK-CARRIED SETSWANA-SPEAKING CHILDREN

**Published:** 2022-01-01

**Authors:** Mariaan van Aswegen, Stanislaw H Czyz, Sarah J. Moss

**Affiliations:** 1 PhASRec, North-West University, South Africa; 2 Faculty of Physical Education and Sport, Wroclaw University of Health and Sport Sciences, Wrocław, Poland; 3 Faculty of Sport Studies, Masaryk University, Brno, Czech Republic

## Background

Setswana children are back-carried by their mother, often deep into their toddler years. Static lower limb investigations of Setswana children rendered differences in the tibiofemoral- and tibial torsion angles between back-carried and non-back-carried children. This leads to the question of whether dynamic differences during gait would be noticed between back-carried and non-back-carried Setswana-speaking children. This study aimed to compare the angles observed at the hip, knee and ankle in the frontal, sagittal and transverse planes, during kinematic gait analyses of back-carried- and non-back-carried children.

## Methodology

Twelve non-back-carried- (mean age=8.00±0.95 years) and 14 back-carried (mean age=8.01±0.73 years) children were selected from a larger (n=691) study. Analysis of hip, knee and ankle joint kinematics, using the Qualisys Track Manager software, were performed in the three cardinal planes, at heel-strike-, mid-stance-, and toe-off gait phases. The angles (outcome variables) were compared with the dependent groups, back-carrying and non-back-carrying, age and sex, using MANOVA.

## Results

A two-way MANOVA was used to evaluate the joint angles observed for back-carried versus non-back-carried children and their respective ages. Back-carrying was not significant, while age was a significant predictor for joint angles, F(1, 17)=209.246, p=0.054, η2=1 and F(1, 17)=8.331, p=0.026, η2=0.986; respectively. The interaction between back-carrying and age was not significant F(4, 34)=1.210, p=0.482, η2=0.911. Some individual significant differences were observed at the knee and ankle joint. A comparison of back-carrying with the knee in the sagittal plane, during the mid-stance gait phase, was significant (p=0.017). Comparisons of age with the ankle; was significant in both the sagittal plane, during heel strike (p=0.016), and in the frontal plane, during mid-stance (p=0.042). A comparison between back-carrying and age with the ankle in the sagittal plane, during the heel strike gait phase (p=0.027), was significant.

## Conclusion

Most Setswana-speaking children are back-carried for the bulk of their toddler years (between 2 to 5 years of age). Relationships between back-carrying and static lower limb measures were previously found but differences were within normal ranges (for example genu valgum), yet none of these relationships translated to the kinematic analysis. Thus, we argue that back-carrying does not pose an orthopedic risk to children.

# Abstract: J3: EFFECT OF 6 WEEKS FUNCTIONAL INTERVENTION PROGRAM ON FMS SCORE AND THE RELATIONSHIP WITH THE SINGLE LEG HOP TEST IN FEMALE NETBALL PLAYERS

**Published:** 2022-01-01

**Authors:** Dimitrije Kovac, Ranel Venter, Zarko Krkeljas

**Affiliations:** 1 Department of Sports Science, Stellenbosch University; 2 Duke Kunshan University

## Background

Functional movement screen (FMS^®^) is currently one of the most widely used pre-participation testing protocols, even though the research studies have reported equivocal results across sports disciplines. Nonetheless, in sports where non-contact lower limb injuries are prevalent as in netball, FMS^®^ may be a valuable tool used alongside other clinical assessment protocols to provide adequate feedback to sport practitioners as the return-to-sport guidelines after an athletic injury. Willingenburg and Hewett (2017) have also indicated that other functional tests such as different forms of the hop for distance test, maybe a more practical and viable addition or even a substitute to FMS^®^.

## Methodology

Convenient sample of 40 elite female university players, randomly divided into control and the intervention group, volunteered for the study. Of the 40 players who were initially tested, nine were excluded from the study either due to withdrawal from the team, or minor injuries. The FMS^®^ score and single leg hop test were measured before and after the six-week training cycle.

## Results

After a six-week intervention there was a significant (p < 0.001) increase in FMS^®^ score for the experimental group, while the control group score remained unchanged. Relative to the relationship between FMS^®^ and hop test, the results show a moderately significant correlation (r=0.48, p < 0.001) between the FMS^®^ total score and single-leg hop test when performed with the dominant leg. Testing on the non-dominant leg also indicated a potentially significant relationship, although statistically not significant (r = 0.34, p=0.06). Similarly, the correlation after intervention remained moderately significant (r=0.36, p<0.05). Athletes with higher FMS^®^ scores had longer jump distances in the SLH test (0.48) which coincides with the results of the study conducted by Willigenburg and Hewett (2017) who reported (r= 0.38 – 0.56).

## Conclusion

The FMS^®^ score in female netball players could be improved by a standardized corrective exercise program. Findings of the current study showed positive correlations between the total FMS^®^ score and SLH test, as well as the HS and SLH when performed with the dominant leg. However, there was no significant improvement in SLH distance after the intervention.

# Abstract: J4: MORPHOLOGICAL CHARACTERISTICS AND THEIR RELATIONSHIPS TO PERFORMANCE IN MALE CROSS-COUNTRY RUNNERS

**Published:** 2022-01-01

**Authors:** Clinton Deon Swanepoel, Dumisane Hlaselo, Adrian Lombard, Andrew Green

**Affiliations:** 1 Department of Sport and Movement Studies, Faculty of Health Sciences, University of Johannesburg, South Africa

## Background

Cross-country running is characterized as high-intensity intermittent and continuously running over surfaces like grass, mud and dirt. Morphological testing relates to tests of the divisions of biology that deals with the systems of living organisms and the connections amongst their structures. Morphological characteristics is used to describe the body shape and proportion of individual bodies. Physiological tests establish how the bodily parts function within the living organism. Physiological characteristics can determine the factors that affect performance and the ability to recover after maximum effort. The aim of this research was to determine the relationship between morphological and physiological characteristics of senior male cross-country athletes in Gauteng province, South Africa.

## Methodology

Forty competitive male cross-country athletes participated in this study. Parameters tested included stature, body weight, seven skinfolds, body fat percentage and lean body mass. The maximal oxygen consumption, running economy and two ventilatory thresholds (VT1 and VT2) were calculated using online assessments. Data were analysed using descriptive statistics (SPSS, v.21). A correlation matrix (Pearson correlation) was calculated between physiological and morphological parameters.

## Results

The results of this study indicated mean values of body weight (63.05 kg), body fat percentage (8.04 %). The mean values for maximum oxygen consumption (VO2max) (63.50 mlO2.kg−1.min−1). The VT1 and VT2 were calculated and at the intensities corresponding to the last point before a first non-linear increase in both VT1 and VT2. The results show a strong positive correlation between stature and mass (r = 0.652, p = 0.01) as well as a weak correlation between BF% and stature (r = 0.382, p = 0.05). There was a positive correlation between BF% and mass (r = 0.575, p = 0.01). There was a weak negative correlation between VO2 and mass (r = −0.368, p = 0.05). The V̇O_2max and VT values did not relate to any physical parameters of this sample.

## Conclusion

These characteristics are generally associated with cross-country runners. This research will serve as a basis for future studies and will provide information on senior male cross-country athletes, which can be referred to by coaches and sports scientists who train athletes during the competition preparation phase.

# Abstract: K1: PSYCHOLOGICAL FACTORS AFFECTING PHYSICALLY DISABLED ATHLETES IN SOUTH AFRICA

**Published:** 2022-01-01

**Authors:** Koketjo Tsebe

**Affiliations:** 1 Unisa, Softball South Africa and SASCOC

## Background

The number of athletes with disabilities participating in organized sports and the popularity of the Paralympic Games is steadily increasing around the world. Many athletes with disabilities agreed that sport is an important tools to affirm their competence and worth. The aim of this study is to evaluate the self-efficacy, athletic identity and motivation of athletes with a physical disability.

## Methodology

The study will adopt a quantitative research approach. It will be an evaluative research design to allow assessing psychological variables under study. A purposive sample of physically disabled athletes from different sporting codes will be invited via their federations to participate in the study. The researcher will select participants based on the pre-defined characteristic, which is gender (male) and age (18 years and above). It is expected that 50 participants (25 females, 25 men) from different sporting codes will participate in the study. Research data will be collected using biographical information and three psychometric tests that measure the psychological variables under study. The biographical section will include variables such as gender, age, sporting code, level of sport participation, disability (congenital, acquired) and type of disability (e.g., spinal cord injury). The three psychometric tests to measure variables under study are: Rosenberg Self-Esteem Scale (RSE), Athletic Identity Measurement Scale (AIMS) and Sport Motivation Scale (SMS). The questionnaire will be available through SurveyMonkey which is an electronic platform for research. Data will be analysed using SPSS version 25 statistical package. The study will abide by the basic ethical standards of social research.

## Results

It can be predicted that physically disabled athletes will report low levels of self-efficacy; physically disabled athletes will identify strongly with their athletic roles, physically disabled athletes will score higher on the sport motivation and there will be a significant relationship between self-efficacy, athletic identity and motivation

## Conclusion

Physically disabled persons are challenged to cope with a wide range of stressors in maintaining meaningful lives. The adjustment depends, in part, on psychological and social factors that promote effective coping with old and new demands.

# Abstract: K2: THE PERCEPTIONS OF PRIMARY CAREGIVERS ON THE SOCIAL SKILLS AND RECREATIONAL ACTIVITIES OF CHILDREN AGED 10–12 YEARS WITH DOWN SYNDROME

**Published:** 2022-01-01

**Authors:** Cara M. Myburgh, Cindy Kriel, Yolanda Stevens

**Affiliations:** 1 FASRek NWU

## Background

Children with Down Syndrome (DS) are known to have fewer responses to social initiations and interactions with peers and other people, which results in difficulties with social relationships. Children with DS are described as being “very sociable” and this is also evident from previous research, where parents of children with DS have different perceptions and views, as some are concerned about their child’s social life and others see them as sociable individuals. Yet, leisure and recreation have a social value which purposefully serve today’s society and the direction of an individual’s life.

## Methodology

This study aimed to determine the primary caregivers’ perceptions of the social skills and recreational activities of their 10- to 12-year-old children with DS. Semi-structured interviews were conducted with 13 primary caregivers, whereafter data was analysed through content analysis.

## Results

Two major categories were identified from the data, each with various themes and sub-themes. The primary caregivers perceived their children to have relative strengths in making friends, as well as non-verbal social skills such as physical proximity and keeping eye contact during a conversation, compared to verbal communication skills in opening and ending a conversation and setting boundaries for self-disclosure. With regard to recreational activities, the primary caregivers mentioned that, at home, their children mainly participate in sports followed by music, drama, fine arts and intellectual/literary activities.

## Conclusion

The children’s behaviour in the majority of social skills was however dependant on various situations, where a certain social skill was sometimes seen as a strength and other times they seem to struggle with the same skill. One of the major areas which primary caregivers identified as barriers to social interaction was their difficulty in speech. Most of children with DS have also never experienced exclusion from participating in leisure and recreation activities.

# Abstract: K3: EFFECT OF LIMITLESS YOU PEAK PERFORMANCE PROGRAM ON THE BRAIN, HEALTH AND SKILL-RELATED FITNESS OF NETBALL PLAYERS

**Published:** 2022-01-01

**Authors:** Peet du Toit, Ralp Hwenjere, Rebekah Janse van Rensburg, Janett Bester

**Affiliations:** 1 Department of Physiology, School of Medicine, Faculty of Health Science, University of Pretoria

## Background

Netball players have to constantly be alert to make quick, precise decisions. Brain fitness is as important as physical fitness in the sport, and an alignment of both would make players particularly competitive compared to the rest. The Limitless You Peak Performance Program (LYPPP) is a highly developed assessment and intervention program, designed specifically to enhance the following three core areas: reducing overall stress, improving cognitive intelligence and the enhancement of overall performance. This program also serves to strengthen the brain-body balance by assessing and training the three core areas of performance, namely, brain performance, health-related fitness and skill-related fitness.

## Methodology

The study was an Interventional pre-post experimental design. The overall current wellness of 30 netball players was evaluated at the start of the study. The LYPPP assessments included brain performance, health-related fitness and skill-related fitness assessments. Following the tests, the participants then participated in the LYPP intervention program. Fifteen intervention sessions were conducted, with each aiming to enhance the brain, health and skill-related fitness of the participant. On completion of the interventions, a post-test was conducted to assess if the intervention employed benefited the performance of the players.

## Results

Significant changes were seen in brain fitness and the players’ overall brain scores. The mean difference in neuro-agility (%) subsequent to the LYPPP intervention was 11.5 ± 13.031 with p = 0.01. No particular change was observed in visual acuity, visualisation, vergence and sequencing after the interventions. The mean letters read for focussing increased by an average of 44 letters read across the team, which was a significant improvement. Tracking and ice-cube tests also showed significant improvement.

## Conclusion

The LYPPP improved brain, health and skill-related fitness in the players. This intervention contributed to the netball team winning the South African tournament. Therefore, due to the positive impact of the LYPPP training, there is potential for this method of training to become more commonly used for the benefit of sports team performance and outcomes.

# Abstract: K4: THE EFFECTS OF THE COVID-19 LOCKDOWN ON PHYSICAL, MENTAL, AND EMOTIONAL PARAMETERS AMONG REGULAR SPORTS PERSONS

**Published:** 2022-01-01

**Authors:** Habib Noorbhai, Amaarah Khan, Ammaarah Patel

**Affiliations:** 1 Faculty of Health Sciences, University of Johannesburg

## Background

The COVID-19 pandemic rapidly spread throughout the world which resulted in a lockdown that was implemented in South Africa on the 26th of March 2020. As a result, it affected the lives of many people, including sportspersons. The purpose of this study was to understand the physical, mental and emotional adaptations that regular sportspersons had to conduct under lockdown regulations.

## Methodology

This was a mixed-methods study in which analytical research methods were employed. A survey (adapted from a Fitness and Wellness Questionnaire) composed on Google Forms was used for this study. This questionnaire included questions about the participants’ (n = 100) physical and mental well-being throughout the lockdown period. Questionnaires were distributed via an online link over social platforms (WhatsApp, Instagram, Twitter, and via email). These answers were exported through an automatically generated Microsoft Excel spreadsheet which was then retrieved by the researchers for analysis. A thematic analysis was also used to identify common themes and stated results of the data. The normality tests used were the Kolmogorov-Smirnov test as well as the Shapiro-Wilk test for statistical analysis, using SPSS (Version 27, IBM). The level of significance was set at p<0.05.

## Results

Participants partook in cardiovascular training, flexibility training, strength training, and bodybuilding exercises (pre-lockdown). During the lockdown, more than 74% of the participants had adequate training space, equipment and time to perform physical activity but more than 43% of these participants experienced a decrease in flexibility, muscle mass, and muscle strength. Exercise was used as a form of stress relief by 77.1% of participants throughout the lockdown. However, participants who used exercise as a form of stress relief continued to experience an increase in stress throughout the lockdown period.

## Conclusion

The outcomes of this study showed that the COVID-19 lockdown had an adverse effect on the overall health and well-being of most sportspersons. Other outcomes included the effects of inactivity, diet and sleep (deprivation) on the body. The extent of such effects could have been further explored by the researchers. Due to social distancing, physiological and morphological measurements could not be conducted which would have yielded further insights into the study population.

# Abstract: L1: EFFECTS OF SOCIAL CONTEXT ON HANDGRIP STRENGTH PERFORMANCE AMONG LESS ACTIVE PEOPLE LIVING WITH HIV

**Published:** 2022-01-01

**Authors:** Laur Gray, Fabienn d’Arripe-Longueville, Maxim Deshayes, Serge S. Colson

**Affiliations:** 1 PhASRec, North West University; 2 LAMHESS, Université Côte d’Azur; 3 PAPSA, Université de Nîmes, APSY-V

## Background

Despite the multiple benefits of physical activity (PA) people living with HIV (PLHIV) remain insufficiently active. As nonexercisers are perceived as less healthy, less active and less strong, it is likely that PLHIV would be subject to the social context they are exposed to. Based on the stereotype threat theory, this study looked to explain the effect of the emerging nonexerciser stereotype on handgrip strength performance among less active PLHIV. We also looked to observe the moderating role of self-efficacy in the relationship between stereotype activation and performance.

## Methodology

Eighteen PLHIV were recruited to participate in two experimental sessions in which both a stereotypical context and self-efficacy were manipulated. Participants performed, in two randomized testing sessions (i.e., high self-efficacy and low self-efficacy), a handgrip task comprising a 15-s fatiguing maximal voluntary isometric contraction (MVC) exercise preceded (Pre) and followed (Post) by 5-s MVCs. In each session, four experimental conditions (i.e., Control 1; Control 2; Stereotypical; Self-Efficacy) were performed.

## Results

A significant performance decrease of the 15-s fatiguing MVC exercise was systematically observed when the nonexerciser stereotype was induced (0.01<p<0.001). In addition, this performance re-increased after a high self-efficacy manipulation (p<0.001). Pre- and post-fatiguing exercise 5-s MVCs were also significantly depressed after the stereotypical condition (0.01<p<0.001) and improved following high self-efficacy manipulation (p<0.001).

## Conclusion

Our results revealed a classical detrimental stereotype threat effect on performance during a handgrip task when the nonexerciser stereotype was activated among less active PLHIV. When high self-efficacy feedback was given, participants’ performance increased significantly whereas no significant difference was observed between the stereotypical and low self-efficacy conditions. These results indicate that self-efficacy can modulate the deteriorating effect of a negative exercise stereotype on task performance in less active PLHIV.

# Abstract: L2: THE EFFECT OF PSYCHO-SOCIAL HEALTH ON THE CORONARY HEART DISEASE RISK INDEX AMONG EMPLOYEES IN A FINANCIAL INSTITUTION OF SOUTH AFRICA

**Published:** 2022-01-01

**Authors:** M Swanepoel, R Labuschagne, J.C Rothmann, S. Rothmann, L.T De Beer, G.L Strydom, M. A. Monyeki

**Affiliations:** 1 Physical activity, Sport and Recreation Focus Area: North-West University; 2 Workwell Research Unit: North-West University

## Background

Psycho-social risks (PSR) appear to be among numerous risks found in the workplace which cause major concern and challenge to occupational health research. The purpose of this study was to describe the effect of psycho-social health on the coronary heart disease risk index (CHDRI) among employees in a financial institution in South Africa.

## Methodology

A cross-sectional design was used, involving an availability sample of 956 employees, aged 20 – 60 years in one of the largest financial institutions in South Africa. Body weight (kg) and height (cm) were measured in accordance with standard procedures and were used to calculate body mass index (BMI). The clinical measurements of systolic blood pressure (SBP), diastolic blood pressure (DBP), total cholesterol concentration (TC) and blood glucose (Glu) were determined according to standard procedures. The CHDRI questionnaire comprises 14 identified coronary heart disease (CHD) risk factors (modifiable and non-modifiable) and were weighted according to the risk to calculate CHDRI and were stratified according to the following cut-points: ≤ 21 = low risk; 22 – 30 = moderate risk and ≥ 31 = high risk. Additionally, psychological ill health (psycho-social stress symptoms, stress-related physical ill health symptoms and burnout risk questionnaire) were used to determine the psycho-social risk status.

## Results

The results indicated that 17% of the employees ran a high risk for CHD and that the risk increased in older employees (40.1%). Additionally, physical ill health and burnout risk appeared to be high, with female employees being mostly affected. Significantly high mean values (χ̄ = 6.50 ± 2.51; χ̄ = 5.15 ± 2.59) and a medium practical effect (d = 0.52; d = 0.56) for psycho-social as well as physical ill health was found in the CHDRI high-risk group compared to the low-risk group. When the ANOVA was calculated the results showed that in all three variables, statistical significant differences occurred between the groups (low- vs. high CHDRI) with psychological stress symptoms F(2) = 16.060, p = 0.001; physical stress symptoms F(2) = 18.165, p = 0.001 and burnout risk F(2) = 3.735, p = 0.025. A significant mean difference (p ≤ 0.05) effect of psycho-social ill health in the low and high-CHDRI groups, with a medium practical significant effect size (d = 0.53) was found. Additionally, physical ill health showed a significant mean difference (p ≤ 0.05) effect on the low and high-CHDRI groups, with medium practical significance (d = 0.57). When comparing the burnout risk effect in the low and high- CHDRI group, a statistically significant difference (p ≤ 0.05) with small practical significance (d = 0.23) was found.

## Conclusion

It can be concluded that the presence of psycho-social ill health among employees in a financial institution in South Africa contributes to a high risk for CHDRI. Given the health implications of these findings for the total well-being of the employees, multiple-component interventions at work sites improve workers’ CHDRI and psycho-social ill health.

# Abstract: M1: PHYSICAL ACTIVITY AND PHYSICAL FITNESS OF OLDER ADULT WOMEN FROM URBAN AND PERI-URBAN AREA OF MAPUTO-MOZAMBIQUE

**Published:** 2022-01-01

**Authors:** Timoteo Daca, Antonio Prista

**Affiliations:** 1 Physical Activity and Health Research Group, Faculty of Physical Education and Sports, Pedagogical University of Maputo, Mozambique

## Background

Physical activity (PA) and Physical Fitness have an important impact on health in the general population including ageing people. Factors that affect the levels include the living environment which has a strong variability.

### AIM

The aim of the present study was to evaluate the relationship between health indicators of older adult women living in urban (UB) and peri-urban (PU) areas of Maputo, Mozambique

## Methodology

A total of 69 older adult women (Age=67.0+6.6 years) participated in this study and were residents in UB (44.9%) and PU (55.1%) areas. Body mass index (BMI) was calculated from height (m^2^) and weight (kg) and participants were classified (Normal; Overweight and Obese) after the standard procedures of evaluation. Moderate to Vigorous Physical activity (MVPA) was assessed by an accelerometer for 7 days. Activities levels (PAL) were classified in low and high 30 min of MVPA. Functional Physical Fitness (FPF) included muscle strength (FM) and cardiorespiratory fitness (ACR) evaluated by the Senior Fullerton test battery procedure (Rikli & Jones 1998). The independent T-test was used for comparisons and the Person correlation to estimate the relationship between the variables.

## Results

No significant statistical differences were found between UB and PU in BMI, MVPA and FPF tests (p>0.05). The prevalence of overweight and obesity was 20.3% and 47.8%, respectively. The subjects have low (18.8%) and higher (75.4%) PAL. BMI was positively correlated with FAT (r=0.827; p<0.001) and negatively correlated with ACR (r=−0.415; p<0.001). The ACR was also negatively correlated with MVPA (r=−0.263; p<0.05).

## Conclusion

The prevalence of cardiovascular health risk factors sense to be evident in older adult women, independent of the residential area of Maputo city.

# Abstract: M2: HABITUAL PHYSICAL ACTIVITY IN CHILDREN AND YOUNG FROM URBAN AND RURAL AREAS OF MOZAMBIQUE

**Published:** 2022-01-01

**Authors:** Gomes Nhaca, Timoteo Daca, Antonio Prista

**Affiliations:** 1 Physical Activity and Health Research Group, Faculty of Physical Education and Sports, Pedagogical University of Maputo, Mozambique

## Background

Studies are showing that Habitual Physical Activity (HPA) of children and young people is drastically reducing in different areas in Mozambique, with repercussions for the quality of life and well-being.

### AIM

The aim of this study was to evaluate and compare the HPA of children and young living in rural and urban areas.

## Methodology

A total of 3 422 children and young (Boys= 49.9%; Girls=50.1%) aged 6 to 17 years from urban centre (URB = 52.3%), a rural area (RUR=23.4%) and an Island (ILA=24.3%) regions were evaluated. The body mass index (BMI) was calculated and the Habitual Physical Activity (HPA) was estimated by the questionnaire (Prista et al 2000). Subjects’ body composition (BC) was classified by BMI (Low, normal, overweight and obese). Levels of activity were compared by activity scores the dimensions of domestic activities (DOM), Games (GAM) Sports (SPO), Walking (WAL), watching TV (WTV) and total activity (TOT). One-way ANOVA and independent T-test were used for comparisons.

## Results

Prevalence of body composition were Low=8.7%, Normal=82.3%, Overweight=5.6% and Obese=3.1%. BMI were higher in URB subjects (URB=18.4±3.5; RUR=16.4±2.3 and ILA=17.3±3.1; p=0.001). Time spent by activity domains was different by region group. RUR subjects performed more time in DOM than URB and ILA (43.7±24.1 vs 12.3±11.2 and 30.3±18.9; p=0.001). ILA subjects play more GAM (37.2±21.1 vs 22.5±14.7 and 24.6 ±18.2; p=0.001) and WALK (14.6 ±3.8 vs 12.3±3.9 and 9.0 ±4.3; p=0.001) than RUR and URB. Time in SPO was higher in URB and ILA than RUR (17.1+14.9 vs 17.4 + 20.1 and 9.2+9.3; p=0.001). The TOT coefficients were higher in ILA, followed by RUR and URB (99.6±44.4 vs 87.7±34.4 and 63.4±31.9; p=0.001). Concerning gender, girls outperform boys in TOT (79.6+39.7 vs 77.5+38.7; p=0.039).

## Conclusion

Patterns and intensities of energy expenditures are strongly influenced by the area of residence of children and youth in Mozambique.

# Abstract: M3: PHYSICAL ACTIVITY LEVELS OF ADULT MALE AND FEMALE FROM A PERI-URBAN AREA OF MAPUTO-MOZAMBIQUE

**Published:** 2022-01-01

**Authors:** Jorge Uate, Ivalda Macicano, Timoteo Daca, Antonio Prista

**Affiliations:** 1 Physical Activity and Health Research Group, Faculty of Physical Education and Sports, Pedagogical University of Maputo, Mozambique; 2 National Institute of Health, Mozambique

## Background

Risk factors for cardiovascular diseases are associated with gender and physical activity. Studies in this topic in peri-urban African settings are scarce.

### AIMS

The aim of this study was to compare the activity levels between adult males and females living in “Polana Caniço”, a peri-urban area of Maputo, Mozambique.

## Methodology

A total of 144 adults (Males= 46.5%; Females=53.5%; Age=31.5+11.9 years) participated in the study. Body mass index (BMI) was calculated from height and weight and Physical activity level (PAL) was estimated by an accelerometer, used for seven consecutive days. Time in sedentary (SPA), light (LPA) and moderate to vigorous physical activity (MVPA) were determined by accelerometer data.

## Results

Percentage of overweight subjects were higher in females (males=13.4%; females=17.1%; p<0.05) as well as obesity (males= 6.0%; females=22.4% ; p<0.05). Average daily time in sedentary activities were significantly higher in females (males=9 894±5 248; females=11 887±9 017; p=0.002), while males spent more time in MVPA (males=42.8±32.3 and females = 18.7±14.0; p=0.001). The percentage of those who accomplished 60 minutes or higher time in MVPA was higher in males (25.4%) than in females( 1.3%).

## Conclusion

Prevalence of overweight, obesity and insufficient physical activity are already at a concern levels being higher in females.

# Abstract: M4: EFFECTS OF THE EMERGING NON EXERCISER STEREOTYPE ON PERFORMANCE ON A FATIGUING TASK IN ACTIVE AND LESS ACTIVE HEALTHY YOUNG ADULTS

**Published:** 2022-01-01

**Authors:** Laura Gray, Maxime Deshayes, Serge S. Colson, Fabienne d’Arripe-Longueville, Corentin Clément-Guillotin

**Affiliations:** 1 PhASRec, North West University; 2 PAPSA, Université de Nîmes, APSY-V; 3 LAMHESS, Université Côte d’Azur

## Background

Perceptions of active and inactive people are conveyed by society and a nonexerciser stereotype has emerged. Past research has focused on information formation with nonexercisers perceived, by both exercising and non-exercising people, as less healthy, less energetic, unfit, less strong and weaker. We know, however, that stereotype threat can contribute to the underperformance of individuals belonging to a range of negatively stereotyped groups and in several domains. Furthermore, fatigue is an important indicator of performance. The present study sought to observe the effect of the nonexerciser stereotype on performance in a fatiguing task.

## Methodology

We used physical activity questionnaires to recruit and compare 13 male nonexercisers and 13 male exercisers’ performance on the fatiguing task, consisting of executing intermittent contractions until exhaustion. Participants completed a familiarisation session as well as stereotypical and nullified sessions.

## Results

A significant condition main effect (i.e., nullified and stereotypical) was shown, F(1, 24) = 40.42, p < .001, η2 = .63. Indeed, this condition main effect was contrary to classic stereotype threat effects, as nonexercisers under stereotype threat improved their performance as revealed by increased time to exhaustion on the fatiguing task. Exercisers also increased their performance when under stereotype threat, in line with the stereotype lift effect. A significant condition main effect also appeared for the rate of perceived exertion (RPE) slope, F(1, 24) = 6.88, p = .015, η2 = .23. The RPE increase was greater in the nullified condition as compared to the stereotypical condition.

## Conclusion

These valuable results observed for rate RPE allow to suggest that RPE could be a mechanism through which performance on a fatiguing task may be affected.

Furthermore, this study is encouraging, in that it revealed that nonexercisers’ performance on a prolonged fatiguing task was not decreased by the negative nonexerciser stereotype.
